# Genomic and metagenomic survey of microbial carbonic anhydrase genes reveals novel clades, high diversity, and biome specificity

**DOI:** 10.1093/ismeco/ycag054

**Published:** 2026-03-11

**Authors:** Mario E E Franco, Esther Singer, Simon Roux, Laura K Meredith, Jana M U’Ren

**Affiliations:** BIO5 Institute, University of Arizona, Tucson, AZ 85721, United States; DOE Joint Genome Institute, Lawrence Berkeley National Laboratory, Berkeley, CA 94720, United States; DOE Joint Genome Institute, Lawrence Berkeley National Laboratory, Berkeley, CA 94720, United States; BIO5 Institute, University of Arizona, Tucson, AZ 85721, United States; School of Natural Resources and the Environment, University of Arizona, Tucson, AZ 85721, United States; Department of Plant Pathology, Washington State University, Pullman, WA 99164, United States

**Keywords:** carbonyl sulfide (COS), OCS, sulfur cycle, carbon cycle, fungi, algae, enzyme evolution

## Abstract

Carbonic anhydrase (CA) enzymes catalyze the interconversion of carbon dioxide and bicarbonate with an efficiency exceeded only by superoxide dismutase. CA enzymes have evolved convergently in phylogenetically distant organisms, forming eight structurally unrelated classes that share physiological functions involved in photosynthesis, respiration, pH homeostasis, CO_2_ transport, and carbonyl sulfide hydrolysis that play central roles in medicine and the environment. Here, we leverage the recent surge in publicly available genomes and metagenomes to re-examine our understanding of the abundance, diversity, and phylogenetic relationships of the three major CA classes in Bacteria/Archaea and microbial Eukaryotes (Fungi, algae). We recovered a total of 57 218 α-, β-, and γ-CA sequences from 24 184 metagenomes and genomes, including the first putative α-CA from an archaeal species. CA sequences formed 3859 protein clusters (1188 with three or more sequences). Sequences within a cluster were typically taxonomically conserved only at higher levels (i.e. Superkingdom, Phylum). When viewed within a phylogenetic framework, the majority of subclades for each CA class contained CAs representing multiple Superkingdoms, although numerous novel β-CA clades appear unique to Fungi. Queries of CA Hidden Markov models against all public metagenome and metatranscriptome datasets revealed that CA is a ubiquitous enzyme present in virtually all sampled environments. However, CA clusters that were taxonomically conserved also appeared more environment-specific, which may explain high CA diversity. This work represents an important contribution to our understanding of the evolution, diversity, and environmental distribution of an enzyme that is key to life and has broad environmental and industrial applications.

## Introduction

Organisms spanning all domains of life encode carbonic anhydrase (CA) enzymes that catalyze the hydration of carbon dioxide (CO_2_; CO_2_ + H_2_O ⇋ HCO_3_^−^ + H^+^) playing key roles in carbon (C) fixation, respiration, CO_2_ sensing, pH regulation, growth, and reproduction [1–4]. Microbial eukaryotes such as red and green algae (Rhodophyta and Chlorophyta, respectively) use CAs to concentrate CO_2_ around RubisCO, enabling them to photosynthesize in C-limited environments such as blooms [5]. CA activity impacts global biogeochemical cycles by facilitating microbial roles in soil inorganic C formation and influencing isotopic signatures of CO_2_ [6–8] and sulfur (S)-containing gases in the atmosphere (e.g. carbonyl sulfide, OCS, or COS) [9, 10]. Moreover, CA activity may allow organisms to acquire sulfur from the atmosphere (e.g. COS + H_2_O → CO_2_ + H_2_S) (e.g. [11]).

Unlike other enzymes that share a single, common ancestor (e.g. bacterial multicomponent monooxygenase (BMM) family [12]), CA enzymes have evolved independently across diverse organisms [13]. As such, CA represents one of the best examples of the convergent evolution of a common catalytic function in phylogenetically diverse organisms [13]. To date, at least eight CA classes have been described (α, β, γ, δ, ζ, η, θ, ι) [2, 5, 14–16]. Most organisms contain one or more CA classes [17] reflecting both intra- and interclass redundancies [18]. Although maintaining proper levels of CO_2_, bicarbonate, and protons is critical for cellular processes [13, 19, 20], CA may be inactivated and/or lost completely for some organisms inhabiting high-CO_2_ environments [21] or that are obligate, intracellular parasites [20]. In addition to convergent evolution, horizontal gene transfer (HGT) may influence an organism’s CA diversity. For example, organisms living in a marine hydrothermal vent appear to frequently exchange α-, β-, and γ-CA genes via HGT [22].

Understanding the diversity, distributions, and functions of different CA classes and isoforms is important for both medical and environmental applications. CA inhibitors, especially those that target specific CA classes or isoforms, are attractive drug targets for the treatment of human diseases. CA inhibitors are used currently as diuretics, antiglaucoma, anticonvulsant, anti-obesity, and anticancer agents [23–30]. As general CA inhibitors can cause side effects in humans, inhibitors that specifically target bacteria or fungal CA isoforms are needed [17, 31, 32]. In addition, different CA classes/isoforms are associated with different ecosystem functions. In marine environments, phytoplankton upregulate specific CA (e.g. δ-, θ-, and ι-CA associated with diatoms) as a C-concentrating mechanism to compete under low CO_2_ conditions [33–35]. In saline and freshwater wetlands, changes in the expression of β-B-CA with salinity and depth are associated with differences in dark CO_2_ fixation [36]. In soil, the bacterium *Thiobacillus thioparus* and fungus *Trichoderma harzianum* encode a CA enzyme in the β-CA D clade (COSase) that can efficiently hydrolyze COS with relatively low CA activity for CO_2_ [37, 38].

A subset of specific CA classes and isoforms, encoded by diverse microbial organisms, may also drive patterns of atmospheric COS. COS not only directly impacts ecosystem sulfur availability, but is also a potential tracer for photosynthesis in terrestrial ecosystems due to the shared biochemical (CA) and physical (stomatal) pathway of COS and CO_2_ in leaves [10, 39]. As demonstrated by inhibitors (e.g. [40]), soil microbes exhibit significant CA activity and the ability to degrade COS [41–43]. Soil COS–degrading microbes tend to encode for β-D-CA [44, 45], which appears to be highly expressed in soil metatranscriptomes [42]. Fungal CAs appear to be particularly important for soil COS uptake [41, 42, 46], which may explain why nitrogen fertilization treatments that tend to alter fungal diversity also result in reductions in soil COS uptake [47, 48]. Leaf litter, which is typically dominated by fungal decomposers, also has high COS uptake rates [49]. Similarly, CA activity, likely of α-CA from certain algal and bacterial taxa [41, 42], influences the atmospheric budgets of δ^18^O in CO_2_ and H_2_O and is therefore important to consider when using COS as a tracer for large-scale patterns in the C cycle and precipitation [41, 42, 50–56].

Despite the ubiquity and importance of CA, few metagenomic and metatranscriptomic studies have specifically examined the diversity and abundance of CA in the environment. As such, the overall abundance, diversity, and distribution of CA across diverse cultured and uncultivated organisms in terrestrial and marine ecosystems remains largely unknown. Here, we use large-scale public genomic data to perform a systematic assessment of the three dominant CA classes (α, β, and γ) spanning both prokaryotic and eukaryotic microbes. By inferring patterns of CA across genomes and environments, we provide a framework to better understand CA abundance and taxonomic conservation across the tree of life, which links CA with its key physiological, ecological, and environmental roles (e.g. as microbial traits for volatile metabolism [57]) that influence atmospheric C and S budgets.

## Materials and methods

### Database mining, sequence clustering, and Hidden Markov model building

Based on functional annotation of α-, β-, and γ-CA derived from Clusters of Orthologous Groups (COGs) [58] and Protein Families (Pfam), we downloaded putative α-, β-, and γ-CA protein sequences from Integrated Microbial Genomes (IMG) [59], MycoCosm [60], and PhycoCosm [61] in May 2021. We used the IMG tool Genome Function Profile, selecting the *assembled* option for datatype, with three COG IDs: COG3338, COG0288, and COG0663. Three Pfam [62] IDs (pf00194, pf00484, and pf00132) were used to query MycoCosm [60] and PhycoCosm [61]. COG and Pfam IDs were chosen based on previous work by Meredith *et al.* [42] that recovered assembled α-, β-, and γ-CA genes from soil metatranscriptomes using Hidden Markov Models (HMMs) built with the above COG IDs. Since MycoCosm and PhycoCosm lack COG annotations, we use the Pfam IDs associated with the above COG IDs in the Joint Genome Institute (JGI) databases MycoCosm [60] and PhycoCosm (Table S1).

CA sequences were retrieved from 24 184 genomes or metagenome-assembled genomes (MAGs) representing 85 bacterial, 12 archaeal, 6 algal, and 9 fungal phyla. After length filtering (see Supplementary Methods; Fig. S1 flowchart), CA sequences were clustered using MMseqs2 [63] at 50% amino acid (aa) similarity (Figs S2 and S3). Following clustering, we constructed multiple sequence alignments (MSAs) with MAFFT v7.453 (*--auto*) [64] and used HMMER 3.3.2 [http://hmmer.org/] to generate HMMs for CA clusters with at least three sequences. Taxonomic information for each sequence was used to determine the lowest common rank (LCR) of each CA cluster (i.e. the lowest taxonomic level shared by all members of the cluster, based on the program MEGAN [65]).

### Multiple sequence alignments and phylogenetic analyses of carbonic anhydrase

We combined the representative sequence of each CA cluster (provided by Mmseqs2 [63]) with reference CA sequences derived from previous studies [2, 37] and records in UniProt/SwissProt with the highest level of annotation evidence [66] (hereafter, UniProt/SwissProt reference sequences: Table S2). Representative sequences of each CA cluster were aligned to the reference alignment for each CA clade using the *--addfragments* option with MAFFT v7.453 [64]. MSAs were then trimmed using trimAl version 2 [67] with the *--gappyout* option, followed by visual inspection and alignment adjustment in Geneious version 2021.02.02 [68]. The alignments for each CA class initially contained 242 (α-CA), 557 (β-CA), or 506 (γ-CA) sequences. After visual inspection, we removed representative CA cluster sequences that appeared fragmented (shorter than <75% of alignment length) and lacked the characteristic CA metal binding sites [2, 69–71]. This removed 217 γ-CA sequences (42.9% of 506), 5 α-CA sequences, and 29 β-CA sequences. The remaining sequences were re-aligned (237 α-CA, 528 β-CA, and 289 γ-CA sequences) as described above and phylogenetic analyses were performed with IQ-TREE multicore version 1.6.12 [72] using the best-fitting model of sequence evolution selected by ModelFinder [73] and 1000 ultrafast bootstrap (UFBoot) replicates [74]. Nodes with <95% UFBoot support were collapsed [75, 76]. Sequences with exceptionally long branches were excluded (three β-CA and three γ-CA) and phylogenetic analyses were conducted again. CA phylogenetic diversity (PD) (i.e. sum of all the branch lengths) was computed for the entire tree and for only fungal sequences using the R package *caper* version 1.0.1 [77], with randomizations as described previously [78].

### Environmental distribution of carbonic anhydrase

We queried 1188 CA HMMs against 3688 published metagenome and metatranscriptome datasets from JGI IMG [59]. Database hits to CA HMMs were filtered based on a score ≥ 50. The highest-scoring open reading frame (ORF) from a metagenome/metatranscriptome was assigned to a single CA HMM. CA hits were used as a proxy for abundance, scaled by study proportion per ecosystem (P) and taxonomic group abundance (T). Scaled values were log-transformed (LOG((P/T) + 0.00001)) and visualized using heatmaps with the R package *pheatmap* v 1.0.12 [79]. For simplicity, we collapsed the detailed ecosystem categories into seven categories: engineered environment, air, aquatic/marine, terrestrial: other, terrestrial soil, plant-associated, and other host-associated (e.g. insect gut).

## Results

### Variation in carbonic anhydrase distributions, identities, and copy number

**Figure 1:**
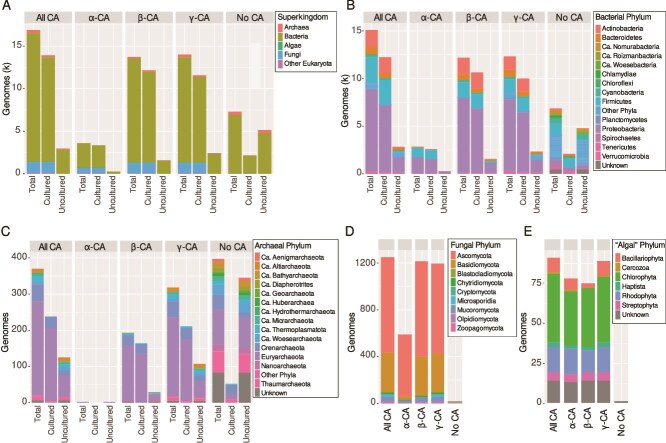
Number of genomes containing CA genes and their taxonomic affiliation by (A) Superkingdom and Phylum for (B) Bacteria; (C) Archaea, (D) Fungi, and (E) algae. In (B), less abundant bacterial phyla were grouped as “Other Phyla” for visualization purposes. For Bacteria and Archaea, the number of CA for cultured vs. uncultured organisms (i.e. MAGs) are shown. Fungal and algal genomes from MycoCosm and PhycoCosm are from cultured organisms, with one known exception (*Acidomyces richmondensis*), which is represented by both a genome and an MAG. Genomes of “algae” in PhycoCosm represent green algae (Chlorophyta) and red algae (Rhodophyta), as well as a diverse assemblage of unicellular eukaryotes, including diatoms (Bacillariophyta), amoeboids (Cercozoa), and haptophytes (Haptista). The term “all CA” refers to combined data from α-, β-, and γ-CA. Also see Table S3. See Fig. S4 for the phylum-level taxonomic information of Bacteria, Archaea, and Fungi.

We compiled 57 218 putative α-, β-, and γ-CA sequences from the JGI databases (Table S3). After filtering potentially misannotated sequences, 54 631 sequences remained (Table 1). Over 70% of genomes/MAGs contained CA proteins. Most of the genomes lacking CA derived from MAGs of uncultured Bacteria and Archaea (Fig. 1, Fig. S4). Seventeen fungal genomes without detectable CAs represented four different phyla (Fig. S4, Table S4). *Guillardia theta* was the only algal genome without detectable CA (Fig. 1, Table S4).

**Table 1:** Summary of CA sequences retrieved, degree of sequence diversity (clusters), copy number variation, and taxonomic conservation at various ranks for each CA class (α-, β-, and γ-CA).

All organisms (16 093 genomes; 8091 MAGs; 24 184 total)	Total CA	Alpha (α)	Beta (β)	Gamma (γ)
CA sequences	54 631	5031	23 906	25 694
CA clusters	3859	885	1359	1615
Sequences in clusters with ≥3 sequences	51 391	4265	22 811	24 315
Clusters with ≥3 sequences	1188	239	468	481
Number of genomes/MAGs (pct of total) with at least one CA	16 892 (70%)	3598 (15%)	13 738 (57%)	14 010 (58%)
Number of genomes (pct of total) with at least one CA	13 932 (87%)	3339 (21%)	12 156 (76%)	11 574 (72%)
CA copies/genome (mean ± SD)	3.4 ± 3.1	1.4 ± 1.6	1.8 ± 1.2	2.0 ± 2.0
CA copies/genome (minimum, maximum)	1, 76	1, 20	1, 29	1, 57
**Bacteria (14 333 genomes; 7630 MAGs; 21 953 total)**				
CA sequences	41 246	3091	19 443	18 712
CA clusters	1634	324	662	648
Sequences in clusters with ≥3 sequences	40 040	2852	18 972	18 216
Clusters with ≥3 sequences (number of unique CA and pct. of all unique)	630 (571 unique)	119 (116 unique; 20%)	271 (246 unique; 43%)	240 (209 unique; 37%)
Number of genomes/MAGs (pct of total) with at least one CA	15 090 (69%)	2850 (13%)	12 191 (56%)	12 324 (56%)
Number of genomes (pct of total) with at least one CA	12 255 (86%)	2593 (18%)	10 638 (74%)	9995 (86%)
CA copies/genome (mean ± SD)	2.8 ± 1.6	1.1 ± 0.3	1.6 ± 0.9	1.5 ± 0.8
CA copies/genome (minimum, maximum)	1, 14	1, 4	1, 13	1, 6
**Archaea (297 genomes; 470 MAGs; 767 total)**				
CA sequences	653	2	219	432
CA clusters	104	1	29	74
Sequences in clusters with ≥3 sequences	593	2	205	386
Clusters with ≥3 sequences (number of unique CA and pct. of all unique)	60 (26 unique)	1 (0 unique; 0%)	17 (10 unique; 39%)	42 (16 unique; 62%)
Number of genomes/MAGs (pct of total) with at least one CA	370 (48%)	2 (0.3%)	193 (25%)	318 (42%)
Number of genomes (pct of total) with at least one CA	245 (83%)	0 (0%)	164 (55%)	211 (28%)
CA copies/genome (mean ± SD)	1.8 ± 0.9	1.0 ± 0.0	1.1 ± 0.3	1.4 ± 0.7
CA copies/genome (minimum, maximum)	1, 7	1, 1	1, 2	1, 6
**Fungi (1268 genomes; 1 MAG; 1269 total)**				
CA sequences	10 228	1003	3785	5440
CA clusters	1248	157	546	545
Sequences in clusters with ≥3 sequences	9163	867	3322	4974
Clusters with ≥3 sequences (number of unique CA and pct. of all unique)	372 (348 unique)	50 (50 unique; 14%)	170 (155 unique; 45%)	152 (143 unique; 41%)
Number of genomes (pct of total) with at least one CA	1250 (98%)	589 (46%)	1215 (96%)	1169 (92%)
CA copies/genome (mean ± SD)	9.7 ± 4.6	1.7 ± 1.2	3.1 ± 2.1	6.2 ± 2.9
CA copies/genome (minimum, maximum)	1, 30	1, 8	1, 18	1, 19
**Algae** ^a^ **(291 genomes)**				
CA sequences	1130	253	204	673
CA clusters	569	163	118	288
Sequences in clusters with ≥3 sequences	576	80	94	402
Clusters with ≥3 sequences (number of unique CA and pct. of all unique)	112 (73 unique)	16 (16 unique; 22%)	28 (16 unique; 22%)	68 (41 unique; 56%)
Number of genomes (pct of total) with at least one CA	91 (31%)	78 (27%)	75 (26%)	89 (31%)
CA copies/genome (mean ± SD)	14.9 ± 8.5	3.6 ± 3.1	3.3 ± 4.0	9.3 ± 5.0
CA copies/genome (minimum, maximum)	1, 66	1, 15	1, 29	2, 32
**Taxonomic conservation by rank** ^b^				
Superkingdom level	95.0	98.7	94.7	93.6
Phylum level	77.7	88.7	79.3	70.7
Class level	59.8	75.7	62.0	49.7
Order level	48.5	68.6	46.4	40.5
Family level	36.5	59.0	32.5	29.3
Genus level	24.8	38.1	23.3	19.8

^a^Genomes of “algae” in Phycocosm represent green algae (Chlorophyta) and red algae (Rhodophyta), as well as a diverse assemblage of unicellular eukaryotes, including diatoms (Bacillariophyta), amoeboids (Cercozoa), and haptophytes (Haptista).

^b^Numbers represent the percentage of clusters with more than two sequences conserved at each taxonomic level. Kingdom level was removed because the Bacteria Superkingdom does not have this taxonomic rank.

CA presence varied by class and taxonomy, with 98.5% of fungal genomes containing CAs, compared to 68.7% of bacterial, 48.2% of archaeal, and 31.3% of algal genomes. However, the percentage of bacterial and archaeal genomes with at least one CA increased to 85.5% and 82.5%, respectively, when restricted to genomes from cultured organisms (Table 1, Table S3B). α-CAs were found in the smallest fraction of genomes (14.9%). Although α-CAs have not been previously detected in Archaea [13, 80], we detected one putative α-CA sequence in each of the two *Archaeoglobus* MAGs [81] (Fig. 1A, Table S3). A higher fraction of genomes, across all taxonomic groups, contained β- or γ-CAs (Table 1).

**Figure 2:**
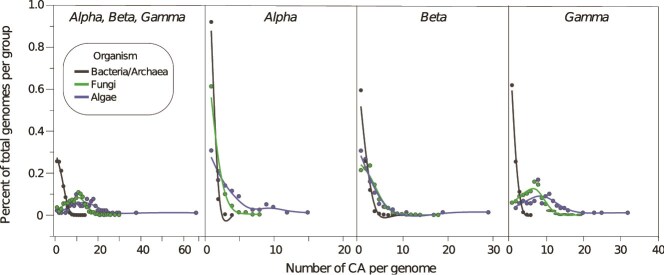
CA copy number variation among different microbial taxonomic groups. The percent of total genomes per taxonomic group with different CA copy numbers for the three CA classes combined (α, β, γ) and for each CA class individually. Points and lines are colored by organismal taxonomy (see legend).

**Figure 3:**
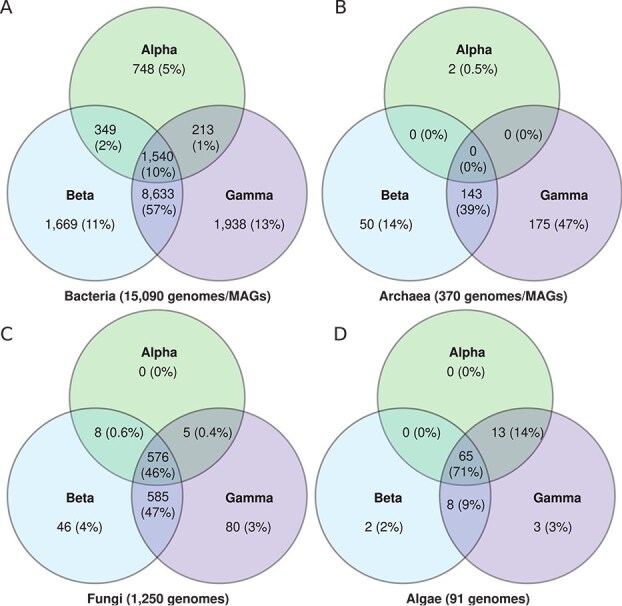
Distribution of different CA classes within microbial genomes. Venn diagrams of the number of genomes (and percentage out of the total number of genomes) that contain different combinations of α-, β-, and/or γ-CA for (A) Bacteria, (B) Archaea, (C) Fungi, and (D) algae. See Fig. 1 for information on the taxonomic affiliation of algal genomes.

CA-encoding genomes averaged 3.4 copies per genome, with algae having the highest mean (14.9 ± 8.5), followed by Fungi (9.7 ± 4.6), Bacteria (2.8 ± 1.6), and Archaea (1.8 ± 0.9) (Table 1, Fig. 2, Table S5). The basidiomycete fungus *Fibulorhizoctonia psychrophila* CBS 109695 (MycoCosm portal: Fibsp1) had the most CA copies (7 α-, 13 β-, and 10 γ-CAs) (Table S5). In genomes with multiple CA classes, a combination of both β- and γ-CA was the most common (Fig. 3). Algal genomes had the highest frequency of all three CA classes, followed by Bacteria and Fungi. In contrast, no archaeal genomes contained all three CA classes and instead were more likely to contain only γ-CA (Fig. 3). No fungal or algal genomes contained only α-CAs.

### Diversity and taxonomic conservation of microbial α-, β-, and γ-carbonic anhydrases

To assess CA diversity among prokaryotic and eukaryotic microorganisms, we clustered all 54 631 CA sequences at 50% amino acid identity, which resulted in 3859 CA clusters that were primarily singletons (Table 1, Fig. 1B, Table S6). Although the number of bacterial genomes and MAGs exceeded eukaryotic genomes 14-fold, the number of CA sequences from Bacteria was only threefold greater than eukaryotic genomes (Table 1). Both Superkingdoms had similar cluster counts after excluding clusters with fewer than three sequences, whereas the smaller number of archaeal genomes resulted in correspondingly fewer clusters (Table 1). CA clusters with three or more sequences were highly conserved at the Superkingdom level, although conservation declined at lower taxonomic ranks (Table 1, Table S7). α-CA clusters were the most taxonomically conserved at all ranks (Table 1). Uncertain rank (i.e. *incertae sedis*) or missing taxonomic information had a negligible impact on LCR assignment.

### Carbonic anhydrase phylogenetic relationships, diversity, and taxonomic conservation

**Figure 4:**
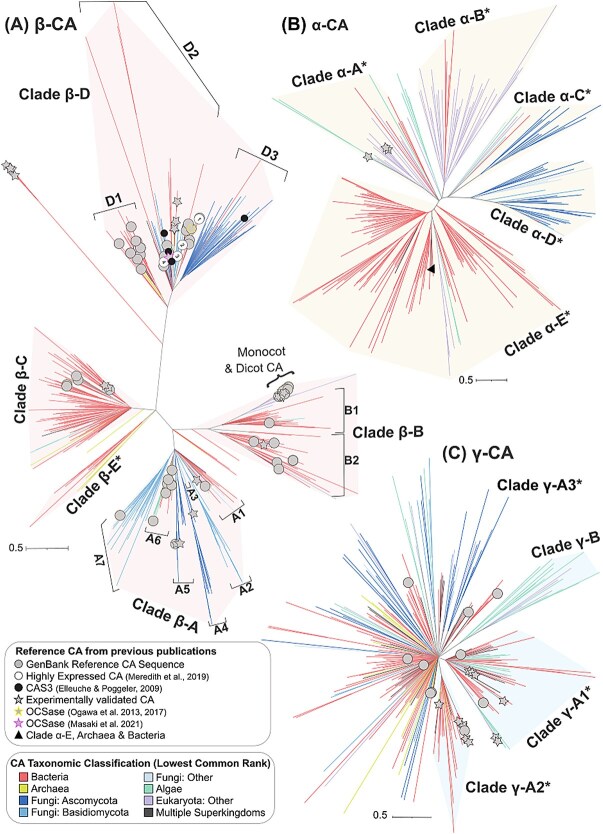
Phylogenetic analyses of α-, β-, and γ-CA cluster representatives reveals novel diversity. Phylogenies of (A) β-, (B) α-, and (C) γ-CAs based on ML analyses. Node support represents 1000 ultrafast bootstrap (UFBoot) replicates. Nodes with <95% UFBoot support were collapsed (see Figs S5–S7 for phylogenies without collapsed branches). Newly described subclades of each CA class are labeled with asterisks (*) (e.g. clade β-E*). Branches are colored according to Superkingdom or Phylum based on the taxonomic lowest common rank (LCR) of each CA cluster (see legend). The scale bar represents the number of substitutions per site. Different symbols indicate significant CA reference sequences from previous studies of CA (see legend). See Figs S8–S10 for each phylogenetic tree with taxon names.

The maximum likelihood phylogenetic analyses of α-CA and β-CA resulted in numerous well-supported clades (UFBoot values >95%), with tree topologies and clades consistent with prior studies. One exception was sequence ZP_00310115, which our analyses placed in the β-C clade, but was previously placed in the β-A by Masaki *et al.* [37] (Fig. 4, Figs S5, S6, S8, and S9). Relationships among most γ-CA clusters could not be resolved with high statistical support (Fig. 4, Figs S7 and S10).

Phylogenetic analyses revealed a large degree of novel CA diversity within each previously described CA class and several novel CA clades (Fig. 4). We identified five new clades of α-CA (α A–E) (Fig. 4B). Additionally, our results support a new β-CA clade (β-E) and three subclades of β-D (β-D1, β-D2, β-D3) (Fig. 4A). Fungal CAs were found in two β-CA clades (β-A and β-D). The β-D2 clade contains previously identified sequences for bacterial and fungal COS-hydrolyzing COSase enzymes [2, 37] and fungal Cas3 enzymes (also in β-D3) [82] (Fig. 4A). Although the phylogeny of γ-CA lacked strong bootstrap support, the resulting maximum likelihood topology supports at least four major clades of γ-CA (Fig. 4C). α-CA had the lowest phylogenetic diversity (PD), whereas β-CA had the highest, but the addition of fungal sequences significantly increased PD across all classes (*P* < .001) (Fig. S11).

### Abundance and distribution of carbonic anhydrase clusters in the environment

We queried 1188 CA HMMs against 3688 published metagenome and metatranscriptome datasets from JGI IMG [59]. CA HMMs had hits in 99.5% of all published studies, and 85% of CA clusters had at least one hit across the 3672 metagenomic and metatranscriptomic datasets that yielded matches. Clusters without hits were primarily assigned to Eukaryota, with β-CAs being the most common (Fig. S12). We found no observable relationship between CA cluster size and the number of hits/cluster (Fig. S13). For α- and β-CAs, clusters that were not conserved at the Superkingdom level occurred in a significantly higher mean percentage of datasets and had a greater mean number of hits to the dataset compared to CAs that were assigned to a single Superkingdom (*P* < .0001) (Fig. S14).

**Figure 5:**
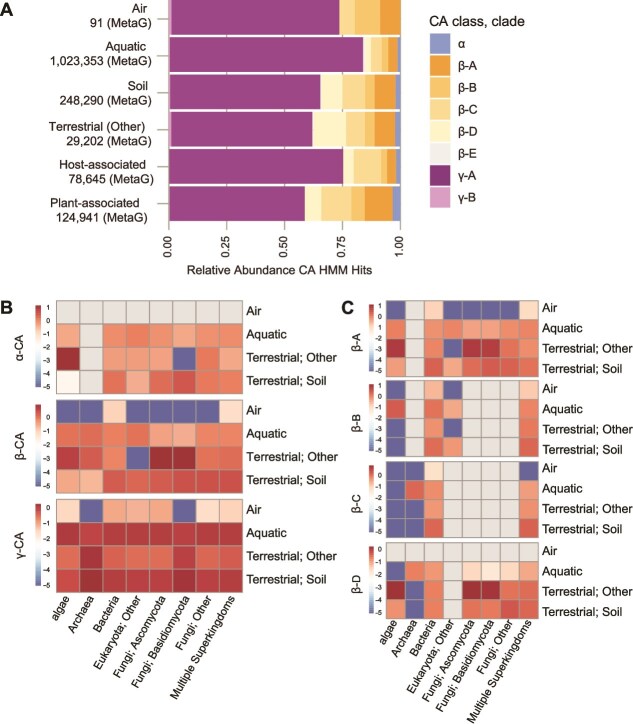
Relative abundance of different CA classes and clades by environment. Data are based on α-, β-, and γ-CA HMM hits to published metagenomic data in IMG. (A) Stacked bar graph of the relative abundance of each CA clade by environment. Numbers represent the number of hits per environment. Clade information follows Fig. 4. Host-associated data are primarily from studies of arthropod gut microbiomes. Heatmaps showing the relative abundance of CA hits across four major environments by taxonomic group (determined by lowest common rank (LCR) assignment) for (B) α-, β-, and γ-CA clusters and (C) four clades of β-CA clusters. We scaled the number of hits by the proportion of studies for the respective ecosystem and the abundance of each taxonomic group to account for potential bias due to different numbers of studies per ecosystem (i.e. a greater number of studies from marine and soil environments) and toward bacteria/archaea whose smaller genomes may be more readily sequenced than eukaryotic organisms. Normalized values were log-transformed for visualization. Gray shading indicates no CA hits.

The highest number of total CA hits occurred in samples from aquatic and soil ecosystems (Fig. 5A, Table S8). Among the three CA classes, γ-A CAs represented >50% of hits in each environment (Fig. 5A, Fig. S15). In contrast, <5% of hits were to α-CAs, and there were no hits to α-CA in air samples (Fig. 5A, Fig. S15). Hits to β-CA clades A–D represented 20%–40% of hits in each environment, but clusters representing β-E CAs had few hits in any environment (Fig. 5A). When data were normalized to account for differences in sample sizes among environments and taxonomic groups, we observed differences in the relative abundance of different CA classes among taxonomic groups and environments (Fig. 5B and C). For example, a higher proportion of hits to fungal and algae β-CAs, especially β-A and β-D CAs, were observed in soils (Fig. 5B and C; see also Figs S16 and S17).

**Figure 6:**
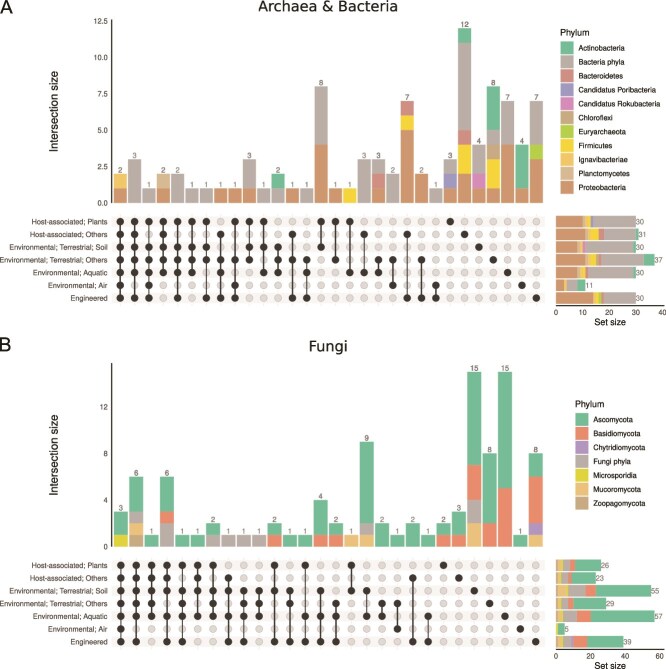
Specificity of CA HMMs to different environmental categories. The intersection of different ecosystems for the ten most abundant HMMs per environment for (A) Archaea and Bacteria and (B) Fungi. Each vertical stacked bar shows the Phylum-level composition and number of CA clusters with that combination of environments (i.e. intersection). Horizontal stacked bars show the Phylum-level composition and number of CA hits (i.e. set size) for each environment. Phylum designation is based on the lowest common rank (LCR) taxonomic assignment. The most abundant HMMs are listed in Table S9.

The most abundant CA clusters displayed limited overlap among different environments, and the majority of CA clusters appear to be specific to either one or two environmental categories (Fig. 6, Fig. S18, Table S9). Bacterial and archaeal CA from each environment were dominated by CA clusters assigned only to the Phylum level or to Proteobacteria (Fig. 6A). CA assigned to Ascomycota were dominant across all environments, especially for CA HMMs only observed in soils (Fig. 6B). There was no clear association between CA clade and environmental distribution (i.e. HMMs from all CA clades were found in most environments) (Fig. 6; set sizes).

## Discussion

### New insights into expanded α-carbonic anhydrase diversity in Gram-positive Bacteria and the Archaea

Previous studies estimate that γ-CA evolved first ca. 4.2 billion years ago [2], followed by the independent evolution of β-CA; however, the age could not be estimated due to accelerated divergence [2, 83]). α-CA are hypothesized to have evolved more recently, arising ca. 0.5–0.6 billion years ago [71]. Among the previous observations supporting this hypothesis was the fact that α-CA had previously only been found in Gram-negative Bacteria [4, 80, 84] until it was recently reported in the Gram-positive *Lactobacillus rhamnosus* GG [85]. Here, we found that ca. 25% of Gram-positive bacterial genomes contained α-CA. Gram-positive bacterial genomes with α-CA included 63 different genera (e.g. *Listeria monocytogenes, Lacticaseibacillus, Oenococcus oeni, Streptococcus, Enterococcus, Streptomyces*, and *Bacillus*). In addition, although archaeal genomes primarily contained only γ-CAs, we detected α-CA genes in each of the two MAGs of the archaeal genus *Archaeoglobus* obtained from fluids collected deep within the Juan de Fuca Ridge subseafloor [81]—a genus known to contain thermophilic sulfate reducers [86, 87]. To the best of our knowledge, α-CA has never been reported in Archaea [13, 80], and the 11 other *Archaeoglobus* genomes included in our analyses lacked α-CA genes. Thus, while our phylogenetic analyses of *Archaeoglobus* α-CA sequences nested within a Bacterial α-CA clade are consistent with *Archaeoglobus* potentially acquiring α-CAs via HGT from Bacteria, additional analyses are necessary to confirm this finding. While we find α-CA to be the most conserved and least phylogenetically diverse of these CA, our large-scale genomic analysis also uncovers a wider distribution of α-CA in prokaryotes than previously appreciated.

### Organisms exhibit diverse carbonic anhydrase genome content

We found that fungal and algal genomes contained more CA copies per genome on average than bacterial and archaeal genomes. Furthermore, almost half of all fungal genomes contained all three classes of CA, as well as duplications within each class. Greater numbers of CA duplications may reflect larger and more complex eukaryotic genomes [88–90]; however, a relatively high proportion of bacterial and archaeal genomes contained multiple copies of β- and γ-CAs, suggesting that genome size alone does not determine the CA copy number. Similarly, certain CA classes were more frequently found in multiple copies per genome, especially for eukaryotes. For example, in eukaryotic genomes, γ-CA had the most copies per genome, followed by β-CA, whereas α-CA was usually not duplicated.

Although widespread, some microbes appear to completely lack CA. One such example is the syntrophic bacterium *Symbiobacterium thermophilum,* which grows on CO_2_ generated from other bacteria [91]. The fact that genomes of closely related *Clostridia* (Firmicutes) contain CA suggests that *S. thermophilum* may have lost the enzyme as an adaptation to a high-CO_2_ environment. To date, few other CA-deficient organisms have been reported [2, 17], although an analysis of published Proteobacteria genomes found 39 strains lacking CA [21]. Similar to *S. thermophilum*, it is hypothesized that loss of CA in these organisms is associated with a lack of selection for CA due to living in high-CO_2_ environments [21].

Here, organisms without CAs were more common among metagenomic-derived genomes (i.e. MAGS), rather than from genomes of cultured organisms. As most culturing approaches are done in relatively low-CO_2_ conditions (ambient), CA-deficient organisms that require high CO_2_ for growth are not captured [21]. Culture-based studies using higher-CO_2_ concentrations are needed to potentially identify more CA-deficient organisms. Host-associated organisms may be more likely to live in high-CO_2_ environments, facilitating continued growth with the loss or inactivation of CA (e.g. [20]). CA inhibitors are a growing area of interest for antibacterial and antifungal treatments [31, 92], with the idea that they are central to the function of these pathogens [30]. However, our data identified numerous genomes that lack genes annotated as CA in multiple phylogenetically diverse taxa, consistent with repeated loss of CA across lineages. This suggests that some microbes not only continue to survive (e.g. fungal CA knockouts survive under low-CO_2_ conditions but with strongly reduced fitness/growth rates [93]), but may also thrive even when lacking CA. Many single-cell genomes and MAGs correspond to poorly understood taxa, thus the full suite of environmental factors contributing to CA loss is unclear. However, it is important to note that the lack of CA in some organisms in this study may be an artifact of incomplete assemblies or MAG binning issues and additional studies are needed to confirm our results.

Known alternate CA classes do not fully explain the lack of CA in these organisms. Some apparent CA-deficient genomes identified here may encode for one or more of the other five described CA classes (i.e. δ-, ζ-, η-, θ-, and ι-CA). However, the phylogenetic distribution of most of these classes is thought to be highly restricted to mainly diatoms and protozoa, and BLAST searches for δ-, ζ-, η-, and θ-CA resulted in <2% of putative CA recovered for α-, β-, or γ-CA and thus cannot explain the large proportion of genomes observed without a CA. The ι-CA appears more broadly phylogenetically distributed compared to δ-, ζ-, η-, and θ-CA [16, 34] but likewise recovered <1% of the putative CA from the IMG database via a BLAST search and is unlikely to explain the widespread lack of CA observed. Nevertheless, the ongoing discovery of new CA, including the ι-CA, suggests that novel CA and their diverse roles in organisms could still be undiscovered.

### Phylogenetic conservation of carbonic anhydrase is strongest at the subclass level

CA sequences (even within a class) are poorly conserved compared to other enzymes [83, 94, 95], as shown by the high number of CA clusters at 30% and 50% aa similarity. Our phylogenetic analyses revealed many unsupported nodes and polytomies, reflecting high sequence divergence and the challenge for short protein sequences to represent deep evolutionary history. Overall, γ-CA phylogenetic relationships were the most difficult to reconstruct, congruent with previous research that suggests the majority of extant γ-CAs arose from multiple simultaneous divergence events that took place relatively close to the last common ancestor of all γ-CAs [2]. This may indicate an ancient diversification of γ-CA potentially associated with different microbial clades and ecological niches. Interestingly, the CA lineages diverging from the largest polytomy in the phylogeny were found to have LCR assignments representing all major taxonomic groups (Eukarya, Bacteria, Archaea). Since γ-CA is hard to identify based on Pfam and COG information due to its diverse functions, we removed sequences misaligned with validated γ-CA metal-binding sites, reducing the sequences used for γ-CA phylogeny. However, there could also be more flexibility in CA functionality than is currently appreciated. For example, despite the classic understanding of CA as strict metalloenzymes, CA from the most recently described class (ι-CA) retains activity even in the absence of a metal cofactor [96]. Future research is needed to functionally validate the CA enzymatic activity of highly divergent γ-CA, which would assist in the development of better methods to identify γ-CA using sequence-based methods.

Unlike γ-CA, α-CA and β-CA phylogenies had stronger statistical support and well-supported clades. Phylogenetic analyses also showed more genetic changes in β-CA compared to other clades, possibly due to the more recent divergence of the α-class [2, 71]. Previous reconstructions indicate frequent duplications and HGT in β-CA, likely due to its modularity [71, 83]. By including LCR taxonomic information for the CA sequences, we found that the majority of well-supported clades had LCRs representing a single Supergroup. For example, numerous fungal- or bacterial-specific clades were evident in both the α- and β-CA phylogenies (Fig. 4). Our β-CA phylogeny recovered most of the clades introduced by Smith *et al.* [2] and at least two additional clades. Our analyses also suggested some α- and β-CA clusters align with microbial taxonomy, potentially enabling CA diversity estimation via taxonomic profile data [97].

### Fungi and algae are important reservoirs of carbonic anhydrase diversity

Genomic data integration revealed an outsized reservoir of CA diversity in eukaryotic microbes. Fungi had the most CA copies per genome, the inclusion of fungal genomes significantly increased known CA diversity, and fungi were dominant members of new CA subclades. Fungal CA are involved in growth and sexual development [93] as well as CO_2_ sensing, which is a critical capability for pathogenic fungi that experience wide-ranging CO_2_ concentrations [98] and modify their expression of virulence traits accordingly [31, 99]. CA duplication may reflect their diverse roles and cellular locations [82], with varied catalytic activity [93, 100]. Fungal CA abundance rivals that of Bacteria for some CA types and environments, yet their role in ecosystem processes has received little investigation thus far (but see [42]).

Here, we confirm the widespread presence of α- and β-CA in Fungi. While the previous focus in Fungi was on specific β-CA isoforms [82, 92, 101, 102], including those similar not only to plants (Cas1 and Cas2) but also to Bacteria (Cas3) [82], our analyses show that fungal CAs are part of a much bigger picture (Fig. 4) (see also [11]). We also inferred abundant and often duplicated γ-CA genes in fungal genomes, which, to date, have received little attention despite being widespread [31, 82, 101]. However, experimental evidence is lacking to demonstrate the CA function of fungal γ-CAs that are present only in the largely unresolved γ-A3-CA clade. The lack of resolution in this clade may arise from a history of rapid divergence, and the γ-CA evolutionary history may be best reconstructed using protein structures. Understanding the distribution of CA across fungal clades will help inform ongoing efforts to develop CA inhibitors as drug targets for fungal pathogens [31].

Finally, our analysis also revealed that algae contain nearly equal numbers of α-, β-, and γ-CA at a high level of duplication, and these algal CA (alongside other eukaryotic CA) may form novel subclades of CA diversity (e.g. γ-A2). Despite the potential to underestimate contributions of Eukaryota using environmental shotgun sequencing, CA hits to transcriptomic data suggest high expression of algal α-, β-, and γ-CA in some environments that complements the activity of their more specialized CA (e.g. δ-, θ-CA). Holistically representing eukaryotic CA represents an exciting frontier for understanding CA function and ecology in the environment.

### Carbonic anhydrases are widely distributed across environments

Overall, all environments investigated were dominated by γ-CA, followed by a sizable fraction of diverse β-CA and low to negligible levels of α-CA. A previous analysis of 10 soil metatranscriptomes also observed a relatively small fraction of α-CA [42]. However, in contrast to our results, that study found a higher proportion of β-CA compared to γ-CA [42]. One explanation may be that here we describe the abundance and diversity of different CA HMMs in these environments based on all available public data, rather than statistically quantifying transcript abundance for samples extracted and sequenced with the same methods [42]. However, analyses of paired metatranscriptomic and metagenomic samples from two studies (soil and marine) revealed similar relative abundances of CA classes in paired data, suggesting that methodological differences alone do not explain the high abundance of γ-CA we observed across environments. Although our analyses included 481 clusters with at least three sequences, the number of small clusters was similar for all CA classes. This suggests that the abundance of γ-CA we observed is not related to including clusters with fewer sequences (i.e. only clusters with >50 sequences were used in ref. [42]). Thus, rather than methodological differences, we hypothesize that a significant increase in amount of data and different studies in the IMG database since 2017 may explain the differences from prior work.

Interestingly, CA HMMs with the most hits across environments tended to display less taxonomic conservation (i.e. were more frequently assigned to LCR at higher taxonomic level), yet these CA HMMs were typically specific to one or two ecosystems. This suggests that certain CA isoforms may be analogous across different microbial lineages, potentially due to environmental selection for specific CA function. Overall, to enable greater functional inferences of CAs from ‘omics data, additional work is needed to experimentally assess enzymatic activity for the diverse CAs identified here.

### A refined picture of carbonic anhydrase in soil and its influence on the global atmosphere

Our approach contextualizes COS degradation within the diversity of microbial CA and extends the current understanding of the specific genes and organisms driving the global soil sink for atmospheric COS. Specifically, we localize known COS activity to the β-D2-CA subclade, which contained experimentally validated COSase sequences from both Bacteria and Fungi [37, 38]. The β-D2-CA clade also contained the primary CA clusters previously found to be expressed in soils [42], as well as sequences identical to the cab-type β-CAS3 CA described in Fungi [82]. The β-D2-CA clade also included taxa previously associated with CA activity for COS through culture-independent (Ascomycota, Basidiomycota, Actinobacteria, and Proteobacteria [42]) and culture-dependent methods [37, 38, 44, 45, 103–107]. In contrast, the β-D3-CA clade was composed almost entirely of fungal sequences representing Ascomycota, and there was only one bacterial CA cluster. A similar Ascomycota-dominated clade was recovered from a recent phylogenetic analysis of COSase-like genes derived from the JGI MycoCosm that implemented different methods (i.e. recovery of similar amino acid sequences using blastp *E*-value 10^−25^ with THIF08 COSase as a query) [11]. Here, we recover multi-kingdom microbial CA diversity that reveals a more balanced role for prokaryotes among β-D diversity. The β-D2-CA clade contains nearly equal representation of prokaryotes and fungi, which differs from the concept of a Basidiomycota-dominated β-D2 clade [11]. We also describe a novel, third β-D-CA clade (β-D1) primarily consisting of prokaryotic sequences. Our analyses of public metagenomic/metatranscriptomic data provide additional evidence for the presence and expression of microbial β-D-CAs in soils and suggest that fungi in the Mucoromycota also may contribute to COS soil fluxes. Moreover, the β-D-CA clade includes proteins with COS hydrolase and carbon disulfide hydrolase activity, which fungi potentially used to obtain atmospheric sulfur when COS levels were higher in the atmosphere [11]. Lastly, our results suggest a higher proportion of β-A-CA and β-C-CA in soil compared to the previous metatranscriptomic analysis [42], which may be due in part to database updates. Although descriptive, the new genomic resources developed here will enable future studies to test specific hypotheses on the role of the β-D2-CA clade in COS consumption in soils.

## Supplementary Material

ycag054_Supplemental_Files

## Data Availability

All data files, including additional files supporting the analyses, are available in FigShare repository (https://doi.org/10.6084/m9.figshare.28156742).
