# Supplementary material for: Genomic and metagenomic survey of microbial carbonic anhydrase genes reveals novel clades, high diversity, and biome specificity
Source: ISME Commun. 2026 Mar 11;6(1):ycag054. doi: 10.1093/ismeco/ycag054 (PMC13098186; doi:10.1093/ismeco/ycag054)
Supplement: ycag054_Supplemental_Files [file ycag054_supplemental_files.zip › SupplementaryMaterials-SupplementaryFigures.pdf]

Supplementary Information

Genomic and metagenomic survey of microbial carbonic anhydrase genes reveals novel clades, high diversity, and biome-specificity

Mario E.E. Franco, Esther Singer, Simon Roux, Laura K. Meredith, Jana M. U'Ren

METHODS

Carbonic Anhydrase Database Mining, Sequence Clustering, and Hidden Markov Model Building. Reference α-, β-, and γ-CA sequences range from 151 to 687 amino acids (aa), while putative CA sequences range from 30 to 5,857 aa (mean ± SD: 255.4 ± 195.0). Due to the potential for mis-assembly, fragmented assemblies, or mis-annotation in genomes and metagenomes, we removed 2,587 sequences with lengths >2 standard deviations of the mean (636.5 aa), including ~650 proteins >1,000 aa in length. We retained shorter sequences (<2 SD) as they contained key CA domains identified by COG and Pfam IDs. The remaining CA sequences were clustered using MMseqs2 [1] at both 30% and 50% aa similarity. Clustering putative CA sequences at 50% identity doubled the number of clusters compared to 30% identity. However, both methods resulted in a similar number of clustered sequences: 51,391 and 53,289 sequences were clustered at 50% and 30% identity, respectively, after excluding clusters with one (singleton) or two (doubleton) sequences. Similar cluster and sequence counts suggest low sensitivity to identity thresholds. All clusters contained sequences from a single CA class, illustrating no cross-grouping between classes. To avoid mixing distinct functions, downstream analyses used clusters from 50% aa identity.

CA sequences were retrieved from 24,184 genomes or metagenome-assembled genomes (MAGs) representing 85 bacterial, 12 archaeal, 6 algal, and 9 fungal phyla. Information on whether a genome was derived from a cultured or uncultured sample (i.e., MAG) was easily available only for genomes retrieved from IMG. Fungal and algal genomes from MycoCosm and PhycoCosm represent culturable organisms, with one known exception (*Acidomyces richmondensis*), which is represented by both a genome and a MAG. Genomes of "algae" in PhycoCosm represent green algae (Chlorophyta), red algae (Rhodophyta), as well as a diverse assemblage of unicellular eukaryotes, including diatoms (Bacillariophyta), amoeboids (Cercozoa), haptophytes (Haptista).

Taxonomic Assignments of CA Representative Clusters. The R packages *taxize* version 0.9.99 [2] and *entrez* version 1.2.3 [3] were used to retrieve the complete taxonomic classification for each CA sequence based on its NCBI taxonomy identifier (TaxId). Taxonomic information for each sequence was used to determine the lowest common rank (LCR) of each cluster (i.e., the lowest taxonomic level shared by all members of the cluster) based on the method used by the program MEGAN [4]. For example, clusters that contain sequences from closely related taxa are assigned taxonomic ranks near the tips of the NCBI tree, whereas clusters with sequences from more distantly related taxa are assigned to taxonomic ranks closer to the root of the tree (i.e., "higher" taxonomic rank) [4]. We used the Medical Subject Heading [5] term "Gram-Positive Bacteria" to obtain a list of such bacteria and examine their representation in the CA dataset.

Phylogenetic analyses of CA clades. Initial phylogenetic analysis of α-, β-, and γ-CA included 242, 553, and 504 sequences, respectively, representing either reference CA sequences with experimental validation (reference sequences listed in Table S2) or representative sequences from each non-singleton or non-doubleton CA cluster (i.e., cluster representative sequences). We removed representative cluster sequences that did not align to the metal-binding residues [6–9], and/or did not encompass at least 75% of the core CA alignment (i.e., the region of the MSA containing >95% of the sequences). The removal of 257 CA cluster sequences (220 representing γ-CA) substantially decreased the number of γ-CA sequences included in phylogenetic analysis (i.e., from 504 to 284 γ-CA sequences). Phylogenetic trees from IQ-TRee were visualized with the Interactive Tree Of Life (iTOL) version 5 [10], Evolview version v3 [11], and T-BAS version 2.1 [12].

RESULTS

Taxonomic conservation of CAs among different CA classes and clades. In addition to fungal-specific β-D subclade (β-D3), β-D1 subclade contained only CA from Bacteria and Archaea, whereas β-D2 contained a greater taxonomic diversity of CA (Fig. 4a). Multiple β-A subclades appear specific to either Basidiomycota (β-A2, A7), Ascomycota (β-A3, A4, A5), or non-dikaryotic fungi (β-A3), which were interspersed with bacterial and algal CA in subclade (β-A6). Fungal CA was also found in subclade β-A1 with CA from Bacteria, Algae, and other Eukaryotes. The β-E clade is comprised of CA clusters with LCRs assigned only to Archaea and Bacteria, similar to its sister β-C clade (Fig. 4a). The β-B clade contained three major subclades of CA that were assigned to both eukaryotes and bacteria. Represented by numerous reference

CA, the γ-A1 clade is comprised of CA clusters assigned to Bacteria, Algae, and other Eukaryotes, as well as CA clusters shared across multiple Superkingdoms (Fig. 4c). In contrast, the γ-B clade is comprised of CA primarily from Bacteria (including numerous reference sequences) and Archaea. The γ-A2 clade contains CA assigned primarily to Algae and other Eukaryota. The largely unresolved γ-A3 clade contains the majority of γ-CA clusters representing CA assigned to all Superkingdoms, including subclades that appear specific to Fungi and other eukaryotes (Fig. 4c). For example, the α-A and α-B clades were comprised of CA clusters with the lowest common rank (LCR) assigned to Bacteria, Algae, and Eukaryota (Fig. 4b). The α-C clade contains two subclades: one clade has CA assigned only to Fungi and another has CAs assigned to both Eukarya and Bacteria (Fig. 4b). The α-D clade also is comprised of CA assigned only to Fungi, whereas the α-E was primarily comprised of bacterial CA (Fig. 4b).

Distribution of CA classes across published metaT/G datasets. Numerous CAs were observed in studies of engineered ecosystems derived from cities; however, environmental samples derived from the air registered the fewest number of CA hits (n = 91) (Fig. 5a, Table S8, Additional file 5). On average, α- and β-CA clusters had hits in <5% of the datasets (mean = 1.9% and 4.9%, respectively), whereas on average γ-CAs occurred in 13% of datasets ($P<0.0001$). The majority of CA were infrequently observed across the metagenomic/metatranscriptomic datasets (i.e., <20%); however, a small fraction of CA clusters were observed in >50% of datasets (Fig. S11a). In addition, the frequency distribution of CA classes differed as a function of LCR assignment (Fig. S11b). The mean frequency and mean number of hits for CA clusters in the metaG/T datasets also differed as a function of LCR taxonomic assignment for each CA class (Fig. S12).

Abundance of γ-CA in metaG/T datasets. Our analysis revealed an unexpected abundance of γ-CA across all environments compared to previous results from Meredith et al. [13]. One potential explanation for this discrepancy is the cluster size threshold (i.e., here, we used a minimum of three sequences per cluster, whereas previous analyses used a minimum of 50 sequences [13]). To assess the impact of cluster size threshold on our results, we examined whether the number of clusters with few sequences (i.e., <4) differed between CA classes. We observed similar numbers of smaller-sized clusters for both β- and γ-CA (89 β-CA clusters and 105 γ-CA clusters with <4 sequences), and for both classes, smaller clusters comprised 19% of the total number of clusters/class. We also found a similar number of clusters with >49

sequences for each class (62 β-CA and 57 γ-CA). These results suggest that the abundance of hits to γ-CA HMMs in metaG/T datasets is unrelated to the CA cluster size threshold used.

Potential bias in metaT/G results. One plausible explanation for the over-abundance of HMM hits to bacterial CA is the strong bias in metaG/T sequences toward bacterial reads [14]. In addition, some environments were sampled more frequently than others in the metaG/T database (Table S8). For example, there were only five hits to host-associated environments in metaT datasets compared to >78k hits to metaG datasets for that same environment, which reflects the few metaT datasets from eukaryotic hosts, as these typically only result in host reads. To address these biases, we performed analyses on all HMMs, as well as on only HMMs with the highest number of hits to the metaG/metaT database at IMG per ecosystem category (i.e., the top 10 or 50 most abundant CA). All analyses supported the wide distribution of CA from α-, β-, and γ-classes across most sampled ecosystems, including both aquatic and terrestrial environments (Fig. 6, Fig. S15, Fig. S16). To account for the over-abundance of bacterial/archaeal reads in metaG/T datasets, we also analyzed profiles of prokaryotic and eukaryotic CA separately. In doing so, we found that sampling the top ten and fifty HMMs per ecosystem reproduced the same results for fungal α-CAs and did not substantially differ for fungal β-CAs. However, analysis of the top 50 CA substantially increased the number of HMMs present in all ecosystems except for air (Fig. S14, Table S9).

To assess whether comparisons of metaT and metaG data from different samples would impact our ability to compare CA abundance using each type of data within an environment, we examined two studies with paired metaG and metaT samples and compared relative abundance of each CA class for each method (Fig. S13B). We identified studies with paired samples using publication information supplied by IMG. We filtered samples based on the same publication that included a similar or equal number of metaT and metaG datasets, and confirmed samples were paired by manually searching IMG using the sample IDs for both metagenomic and metatranscriptomic data. In both soil and marine samples, we observed a similar relative abundance of each CA class for each sequence type (Fig. S13B). This suggests that either metaT or G data could be used to examine relative CA abundance in different environments, but also that we should not compare relative CA abundance using metaG and metaT samples from different studies (i.e., differences are likely due to samples). However, analyses with paired samples (Fig. S13B) and with only metaG data (Fig. 5A) both found that samples from aquatic environments have a higher fraction of γ-CA compared to soil samples.

LIST OF SUPPLEMENTARY TABLES

Table S1. CA protein families, their description, and number of CA recovered from IMG/MER, MycoCosm, and PhycoCosm (a), IMG/MER (b), MycoCosm (c), or PhycoCosm (d). Different approaches were used, such as BLAST searches or sequence retrieval using COG, Pfam, KEGG, Interpro, and EC IDs. The values are based on the published genomes as of May 5th, 2021.

Table S2. Reference sequences used in the phylogenetic analyses.

Table S3. Table showing the number of CA sequences per database and group of organisms (a), the number of genomes containing at least one CA per database and group of organisms (b), the number of genomes without CA per database and group of organisms (c), and the number of genomes with a single class of CA, two classes of CA, and the three classes of CA. The values are based on the published genomes as of May 5th, 2021.

Table S4. Fungal and algal genomes without detectable CA genes.

Table S5. Frequency of CA per genome (a), also detailed by database (b), superkingdom (c), and group of organisms (d). The values are based on the published genomes as of May 5th, 2021.

Table S6. Clustering summary, after clustering sequences at 50% (a, b) and 30% (a, c) sequence identity thresholds.

Table S7. Percentage of α-, β-, and/or γ-CA clusters unique to a specific superkingdom (a), kingdom (b), phylum (c), class (d), order (e), family (f), and genus (g).

Table S8. Count of HMM hits to the metaG/metaT database at IMG per environment. (a, b) α-CA HMMs. (c, d) β-CA HMMs. (e, f) γ-CA HMMs.

Table S9. Ten and 50 most retrieved HMMs per ecosystem, after subsampling archaeal and bacterial HMMs (a-f) or fungal HMMs (g-l). HMMs were queried against the metaG/metaT database at IMG.

SUPPLEMENTARY FIGURES

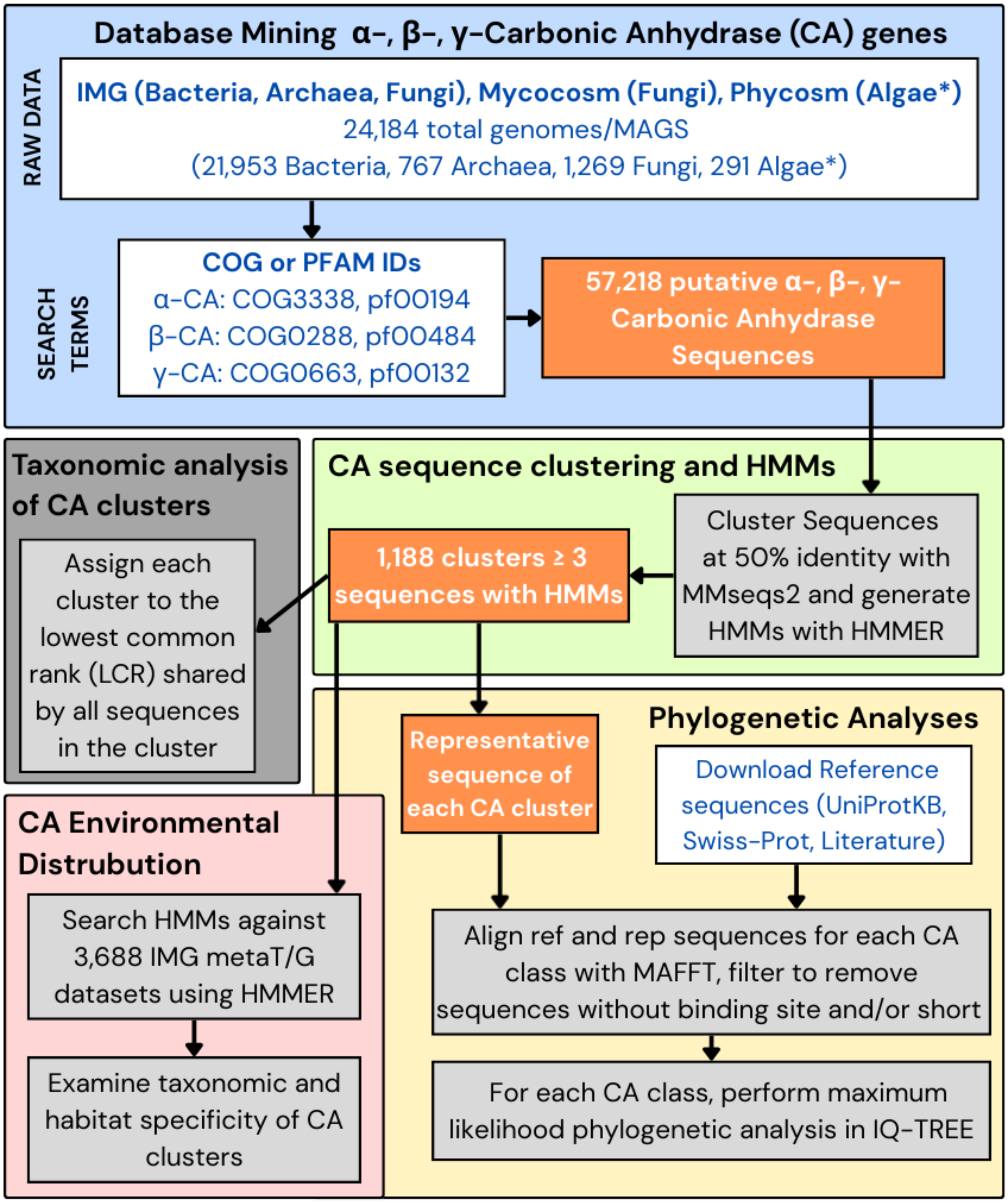

Figure S1. Flowchart of the steps and analytical methods used in the present study,

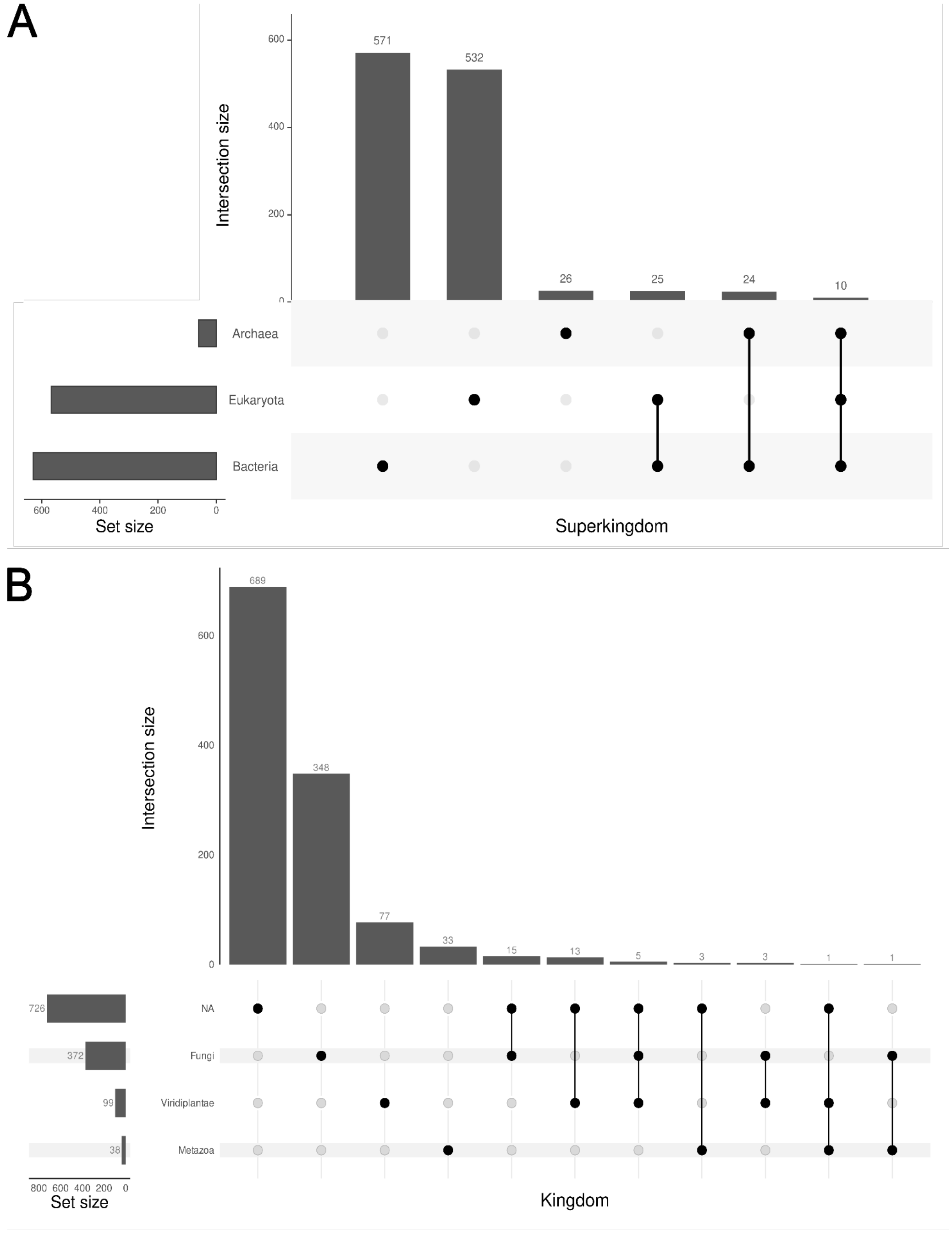

Figure S2. Upset plot illustrating the intersection of different (A) Superkingdoms and (**B**) Kingdoms for Mmseq2 clusters with more than two sequences.

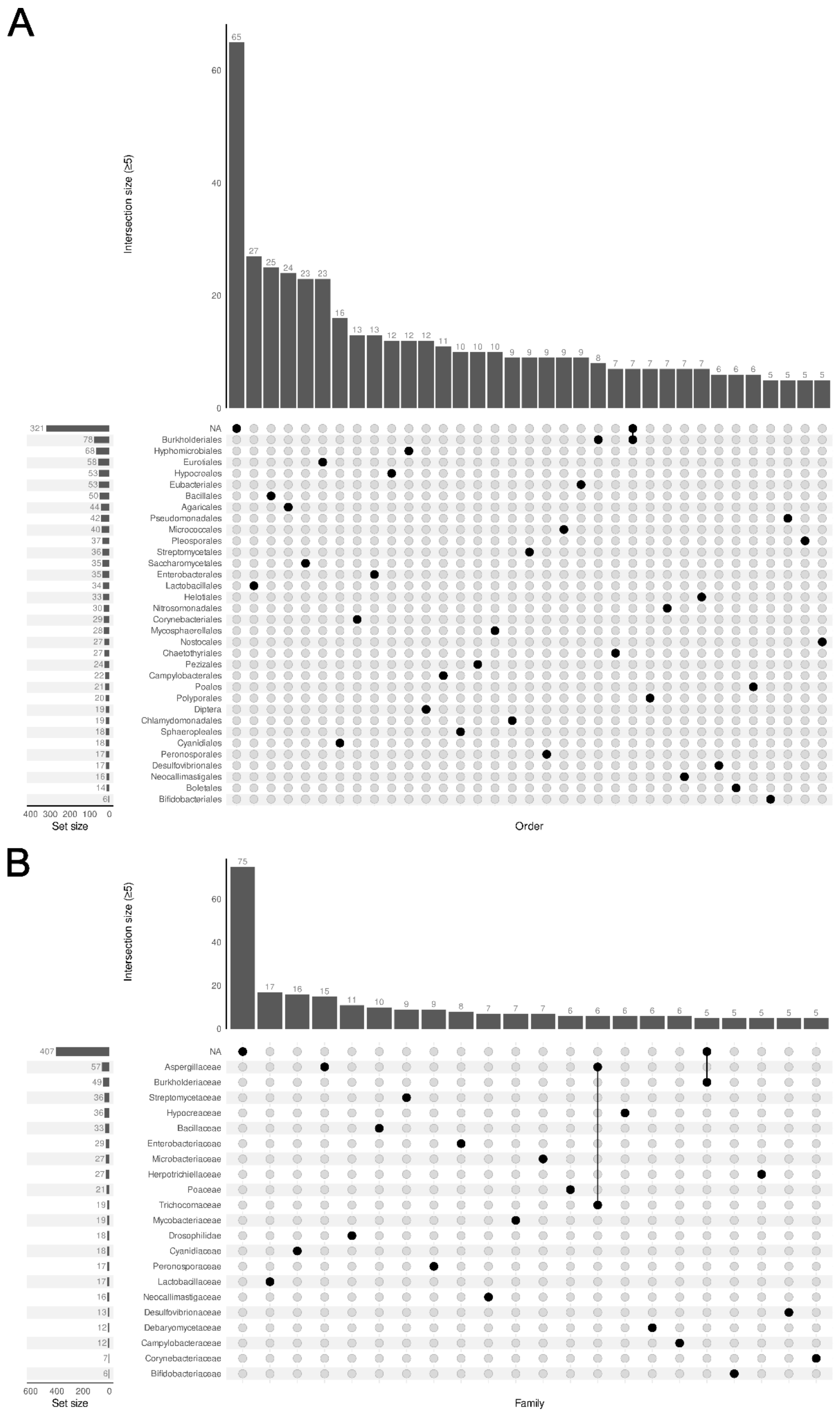

Figure S3. Upset plot illustrating the intersection of different (A) orders and (B) families for Mmseq2 CA clusters containing more than two sequences. Only intersection sizes greater than or equal to five are shown.

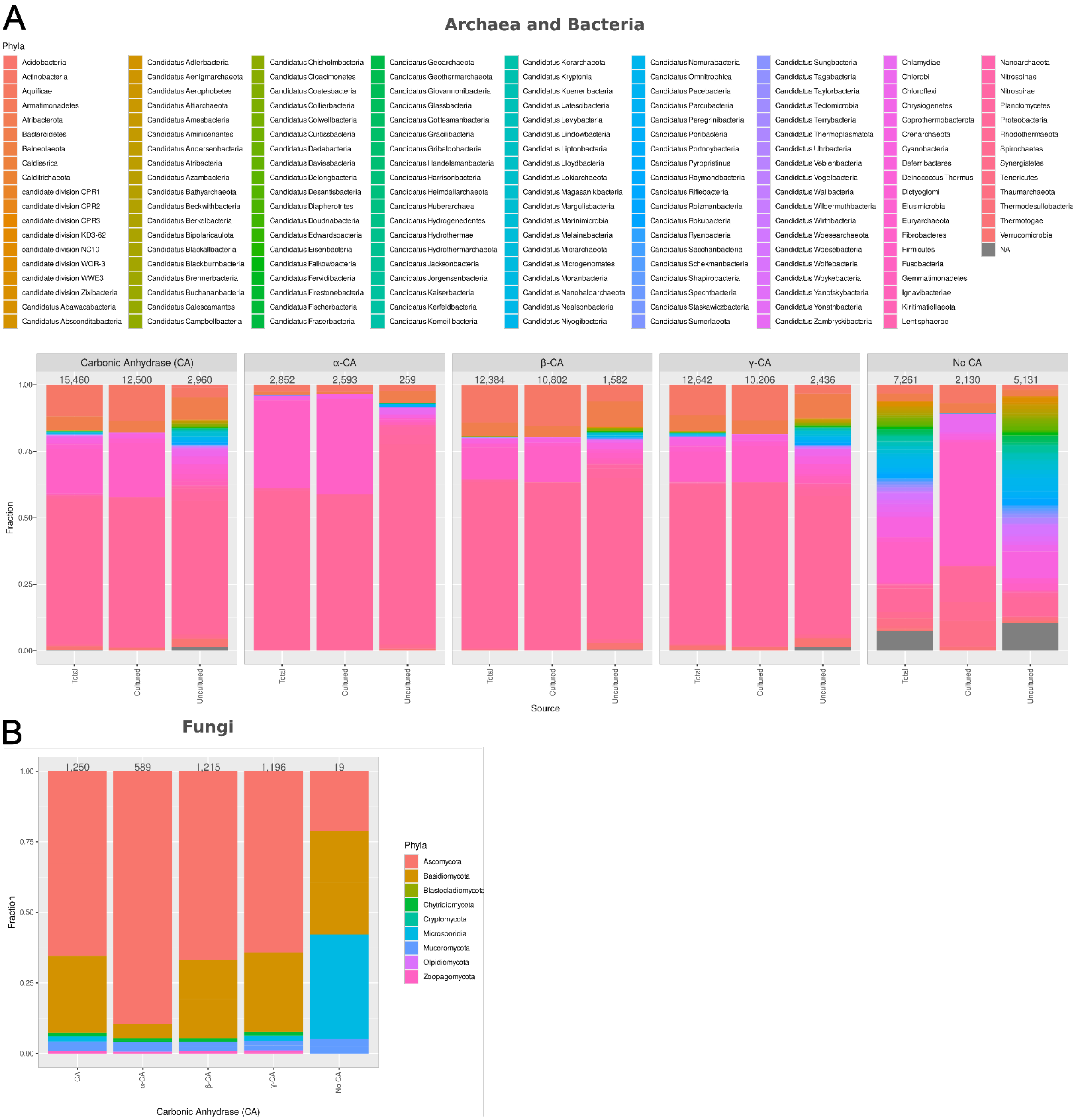

Figure S4. Relative abundance of genomes representing (A) Bacteria and Archaea, or (B) Fungi with and without CA. Genomes of Bacteria and Archaea in IMG are derived from both cultured and uncultured organisms (i.e., metagenome assembled genomes). The number at the top of each bar indicates the number of genomes in the corresponding category. Also see Fig. 1.

α-CA

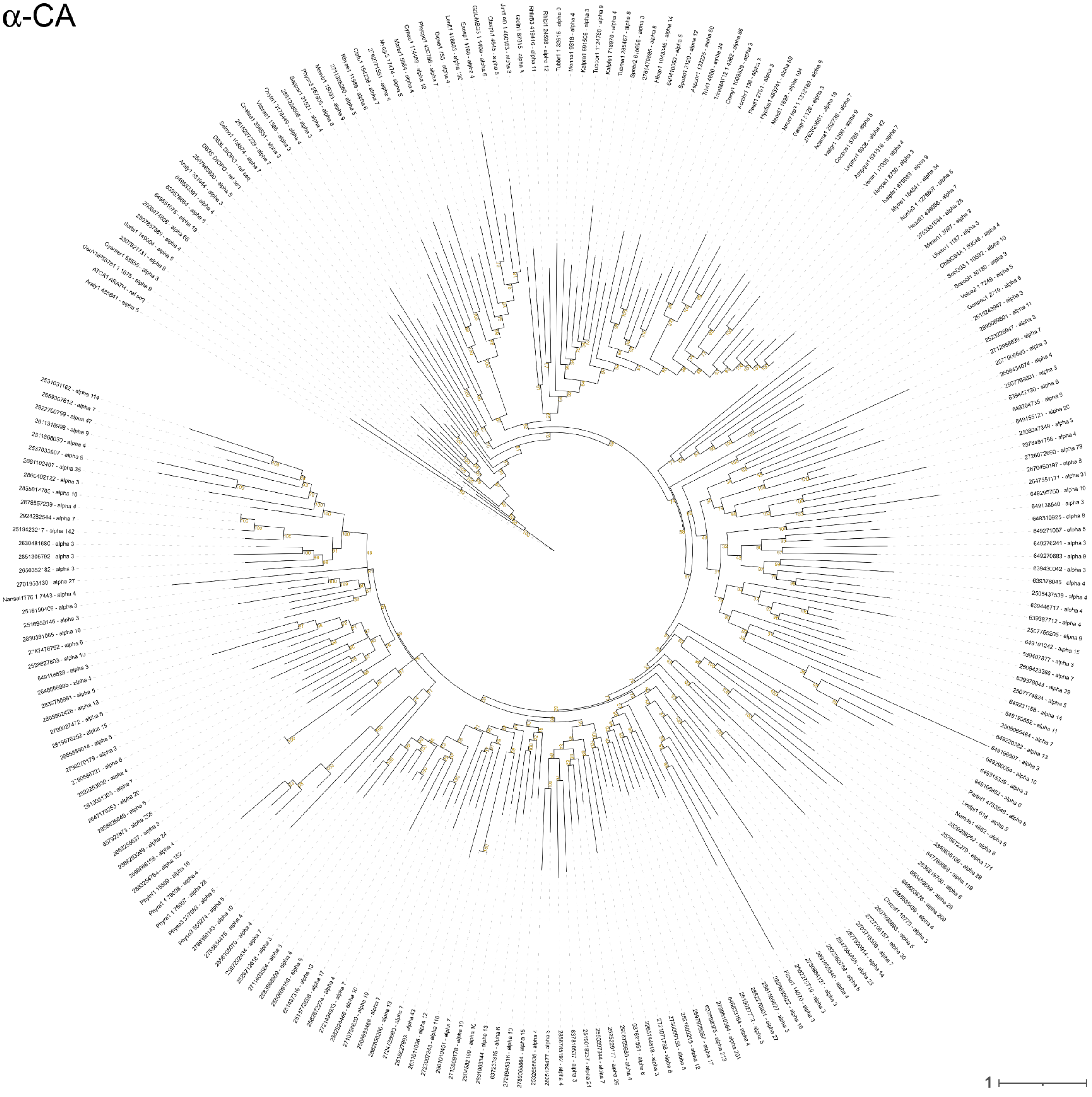

Figure S5. Phylogenetic tree of α-CAs. The phylogeny was reconstructed using the cluster representative sequences and the reference sequences by a maximum-likelihood approach with IQ-TREE multicore version 1.6.12, employing the best-fitting model of sequence evolution selected by ModelFinder. Node support was evaluated using 1000 ultrafast bootstrap (UFBoot) replicates. Node support is indicated in mustard. The scale bar represents the number of substitutions per site.

β-CA

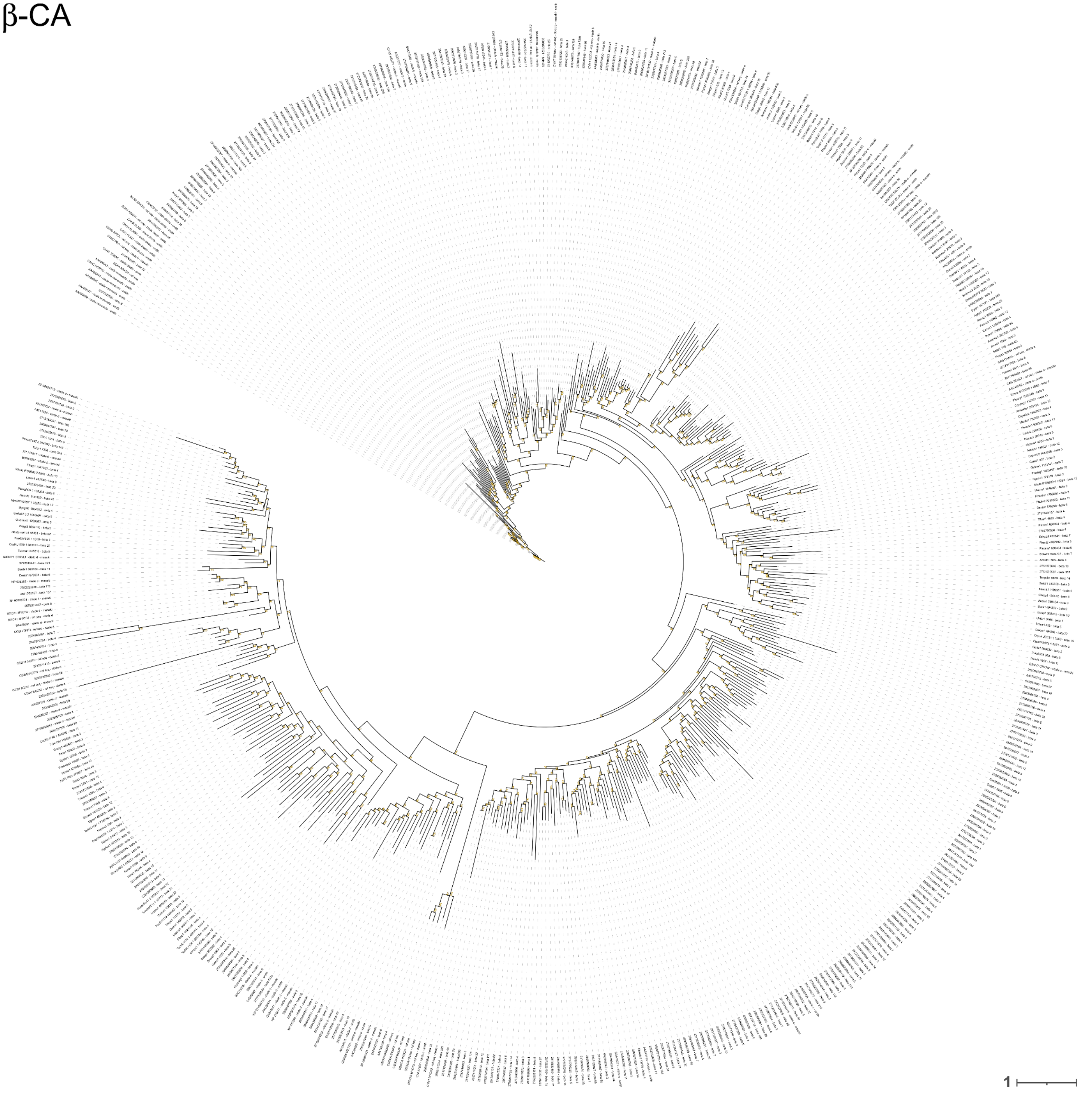

Figure S6. Phylogenetic tree of β-CAs. The phylogeny was reconstructed using the cluster representative sequences and the reference sequences by a maximum-likelihood approach with IQ-TREE multicore version 1.6.12, employing the best-fitting model of sequence evolution selected by ModelFinder. Node support was evaluated using 1000 ultrafast bootstrap (UFBoot) replicates. Node support is indicated in mustard. The scale bar represents the number of substitutions per site.

γ-CA

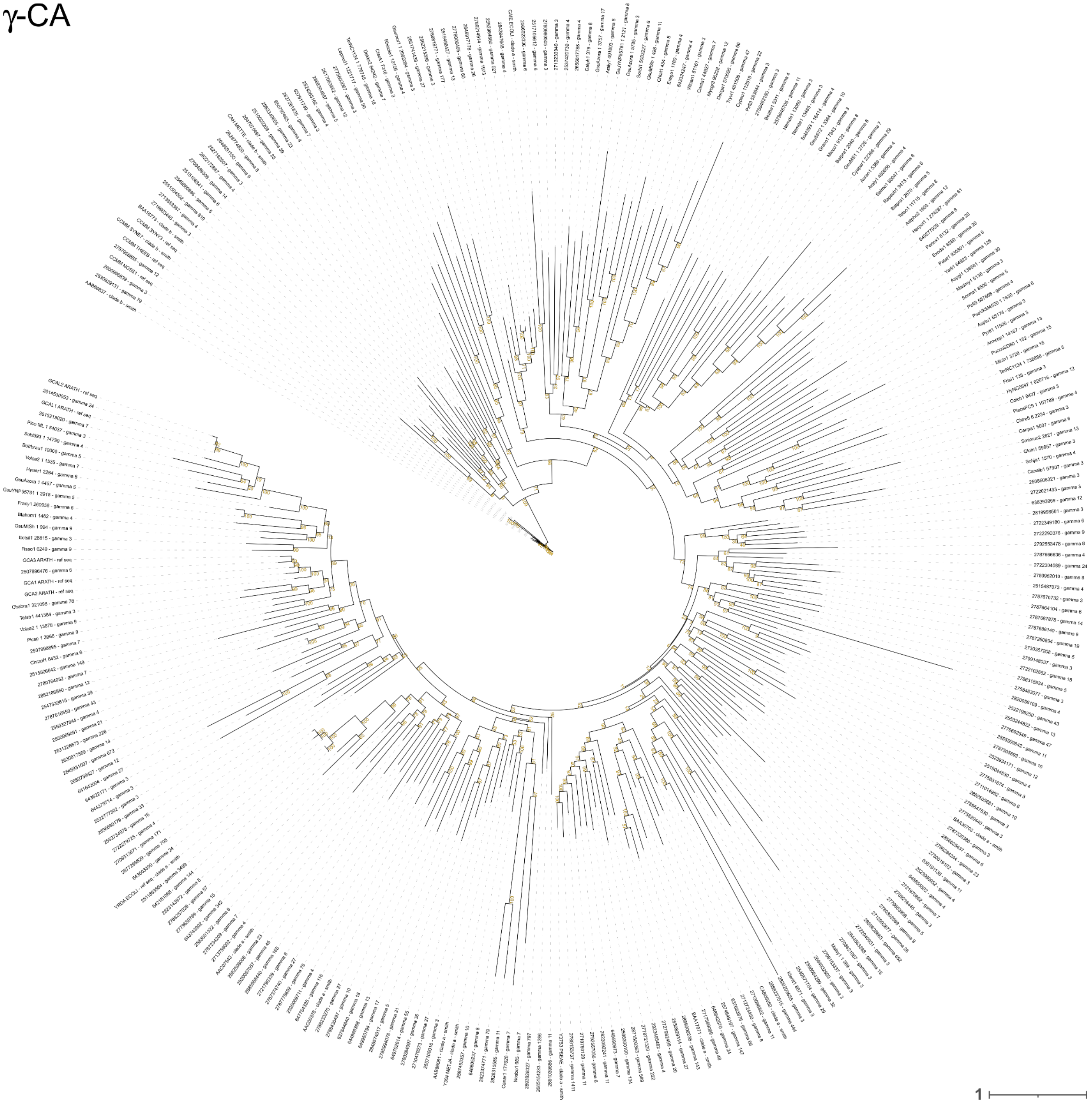

Figure S7. Phylogenetic tree of γ-CAs. The phylogeny was reconstructed using the cluster representative sequences and the reference sequences by a maximum-likelihood approach with IQ-TREE multicore version 1.6.12, employing the best-fitting model of sequence evolution selected by ModelFinder. Node support was evaluated using 1000 ultrafast bootstrap (UFBoot) replicates. Node support is indicated in mustard. The scale bar represents the number of substitutions per site.

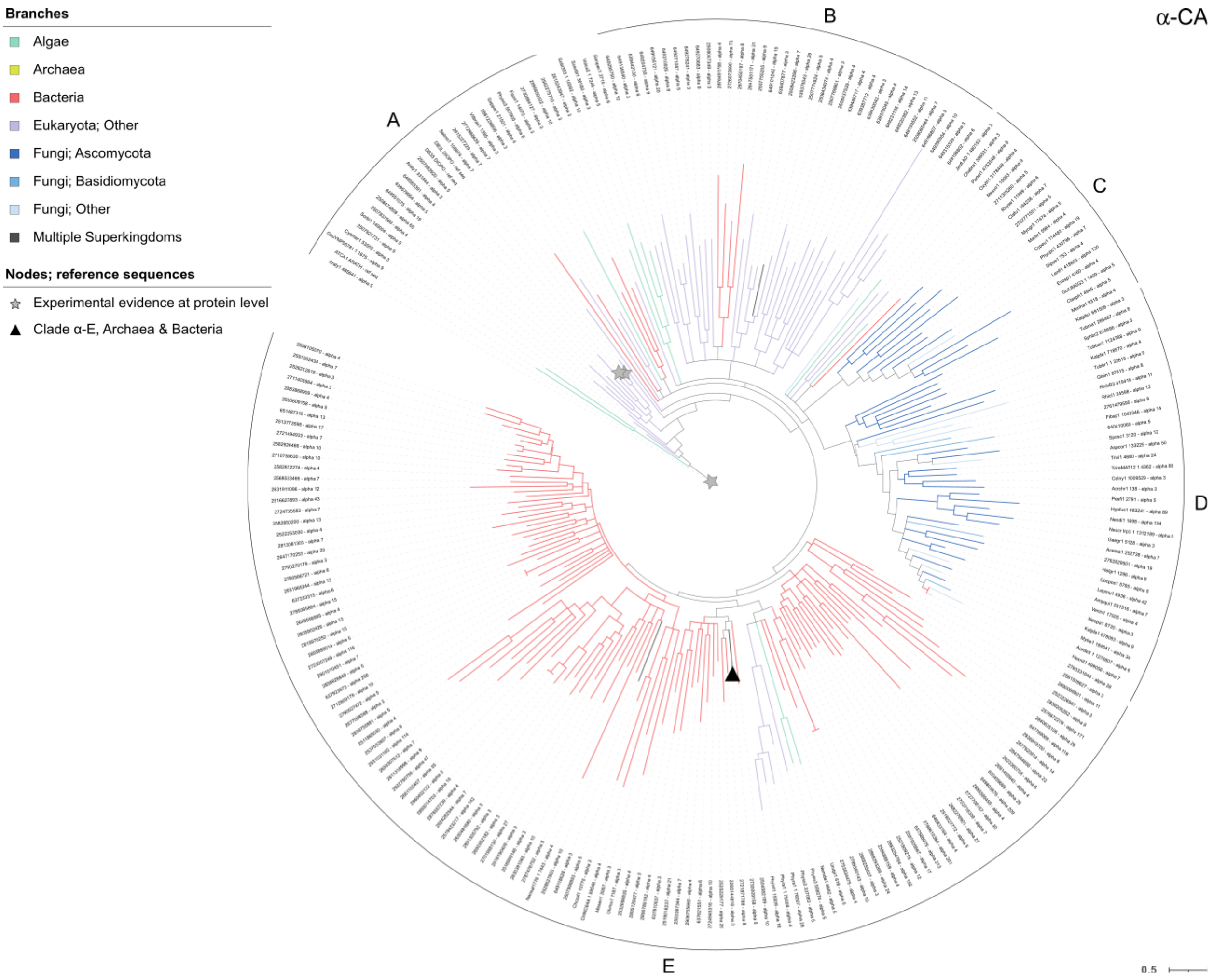

Figure S8. Phylogeny of α-CA after collapsing nodes with low support values. The phylogeny was reconstructed using the cluster representative sequences and reference sequences by a maximum-likelihood approach with IQ-TREE multicore version 1.6.12 employing the best-fitting model of sequence evolution selected by ModelFinder. Node support was evaluated using 1000 ultrafast bootstrap (UFBoot) replicates. Nodes with less than 95% UFBoot support were collapsed. The scale bar represents the number of substitutions per site. Branches are colored according to the cluster representative taxonomy assigned by the lowest common rank-like approach. The stars indicate reference sequences labeled as 'Experimental evidence at protein level' in UniProtKB.

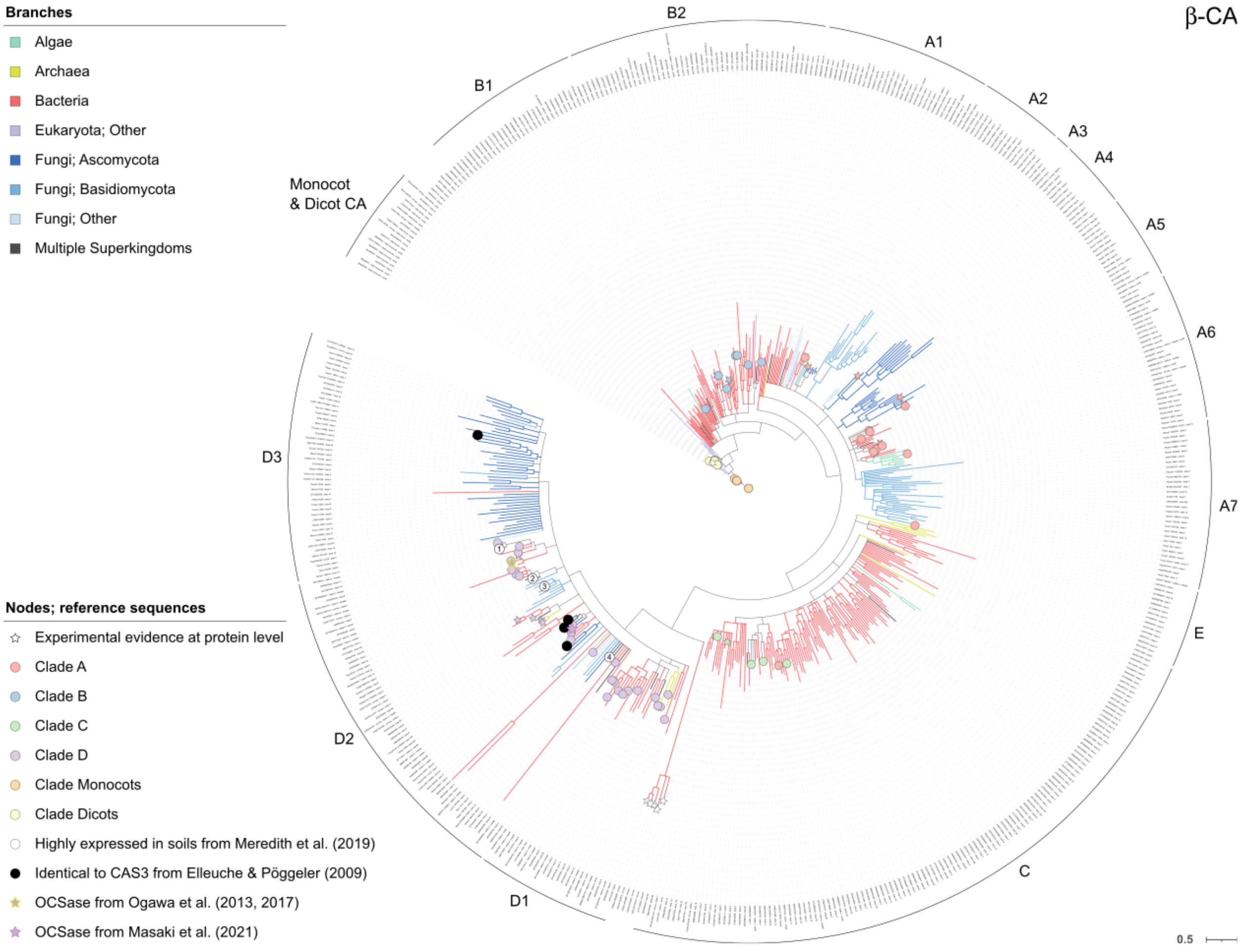

Figure S9. Phylogeny of β-CA after collapsing nodes with low support values. The phylogeny was reconstructed using the cluster representative sequences and reference sequences by a maximum-likelihood approach with IQ-TREE multicore version 1.6.12 employing the best-fitting model of sequence evolution selected by ModelFinder. Node support was evaluated using 1000 ultrafast bootstrap (UFBoot) replicates. Nodes with less than 95% UFBoot support were collapsed. The scale bar represents the number of substitutions per site. Branches are colored according to the cluster representative taxonomy assigned by the lowest common rank-like approach. Reference sequences are indicated with a symbol at the edge of the tip, which is colored based on the clade assignment Smith et al. (1999) and Masaki et al. (2021). The stars indicate reference sequences labeled as 'Experimental evidence at protein level' in UniProtKB.

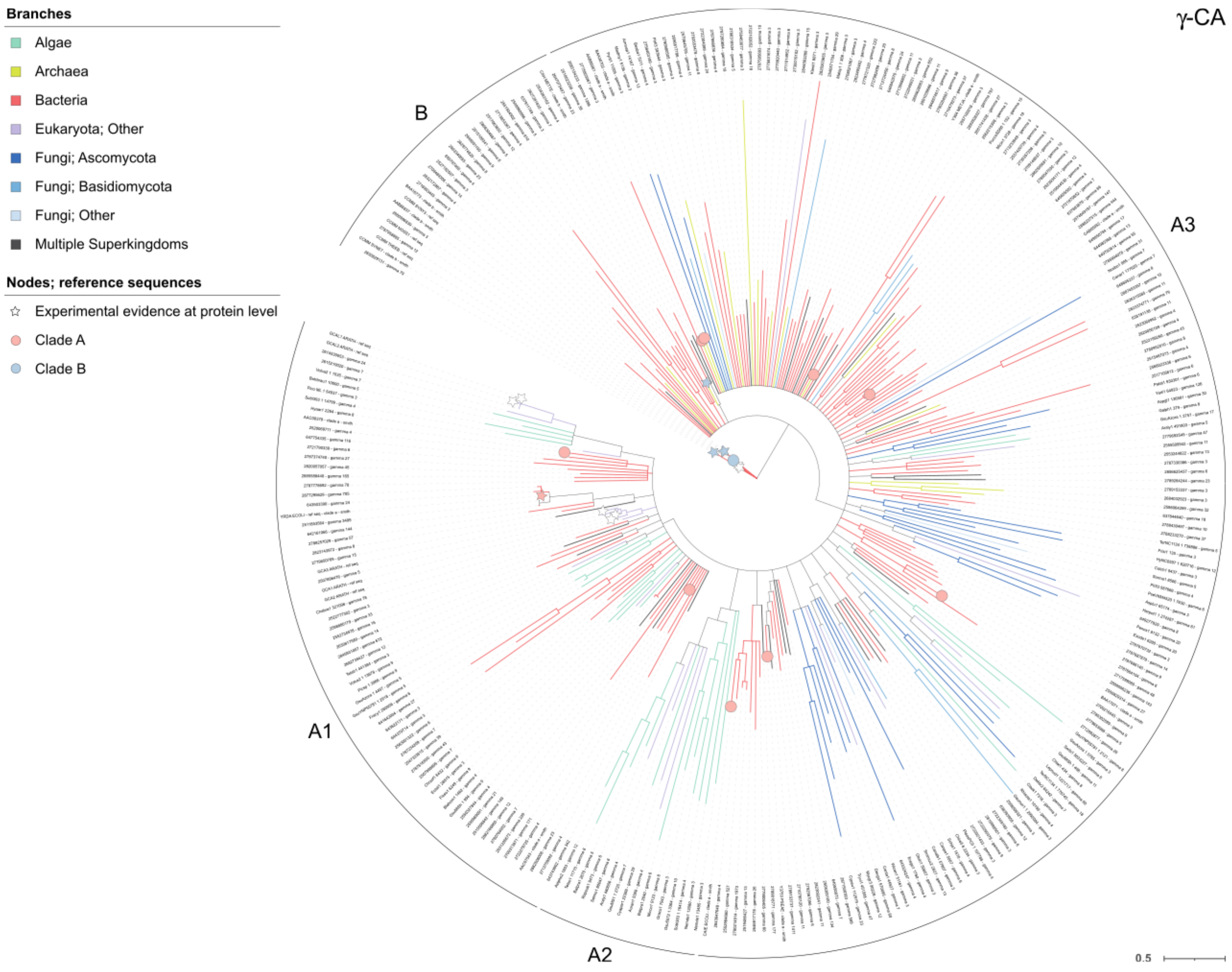

Figure S10. Phylogeny of γ-CA after collapsing nodes with low support values. The phylogeny was reconstructed using the cluster representative sequences and reference sequences by a maximum-likelihood approach with IQ-TREE multicore version 1.6.12 employing the best-fitting model of sequence evolution selected by ModelFinder. Node support was evaluated using 1000 ultrafast bootstrap (UFBoot) replicates. Nodes with less than 95% UFBoot support were collapsed. The scale bar represents the number of substitutions per site. Branches are colored according to the cluster representative taxonomy assigned by the lowest common rank-like approach. Reference sequences are indicated with a symbol at the edge of the tip, which is colored based on the clade assignment Smith et al. (1999). The stars indicate reference sequences labeled as 'Experimental evidence at protein level' in UniProtKB.

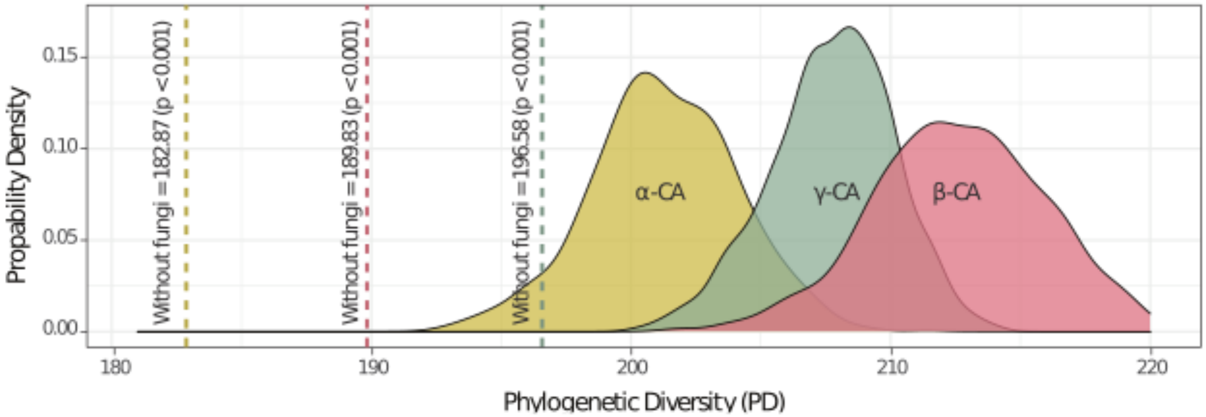

Figure S11. Fungal CA significantly increase the known phylogenetic diversity of CA in each class. Probability distribution of the phylogenetic diversity of each CA class based on maximum likelihood phylogenetic analyses (i.e., Fig. 4; Figs. S4-S9). The discontinuous line indicates the value of phylogenetic diversity (PD) obtained when fungal sequences were excluded from analyses. P-values were calculated using 1000 random subsets of non-fungal clusters in such a way that the sample size equals the total number of non-fungal clusters.

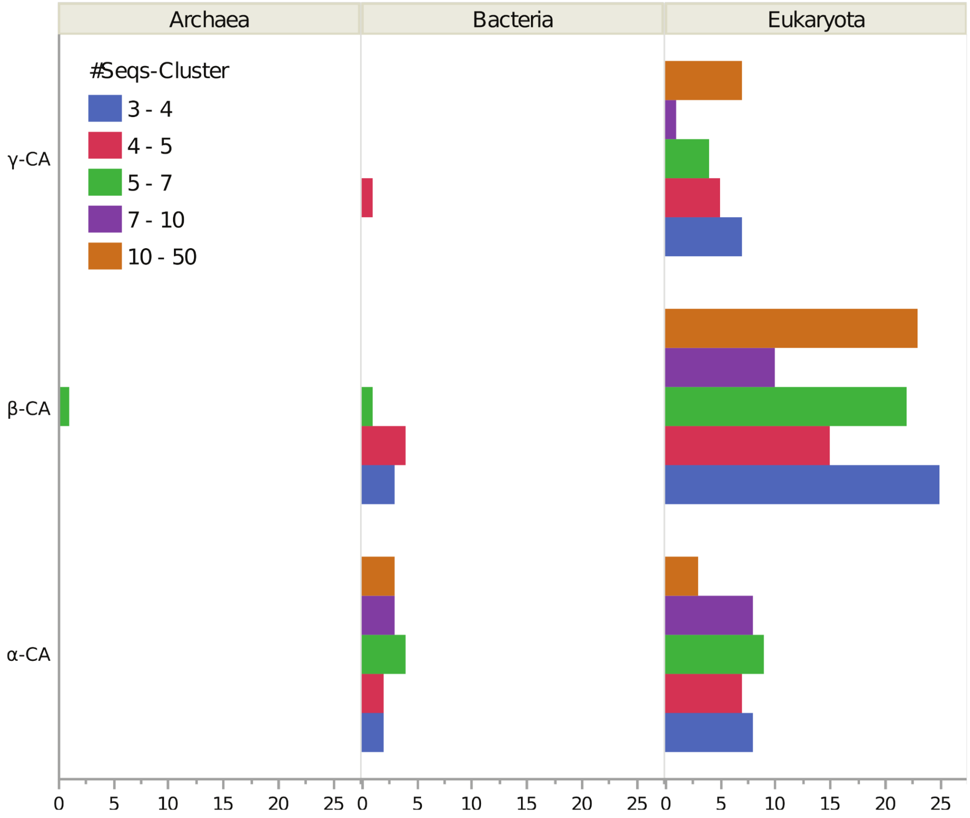

Figure S12. Histogram of cluster size for α-, β-, γ-CA clusters with HMM that did not match any of the CA sequences obtained from previous metagenomic and metatranscriptomic studies. Bars are colored by the number of sequences in each CA cluster.

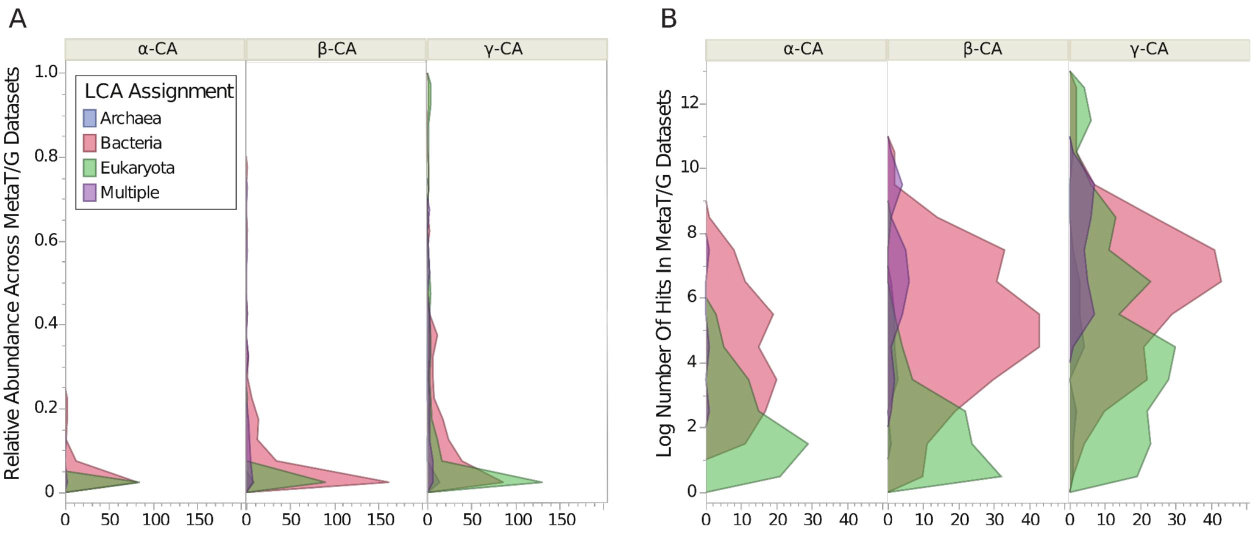

Figure S13. Frequency distribution of the (**A**) relative abundance of each CA class out of 3,672 metagenomic/metatranscriptomic datasets and (**B**) log number of total hits for each CA class. Colors indicate the LCR taxonomic assignment of each CA cluster.

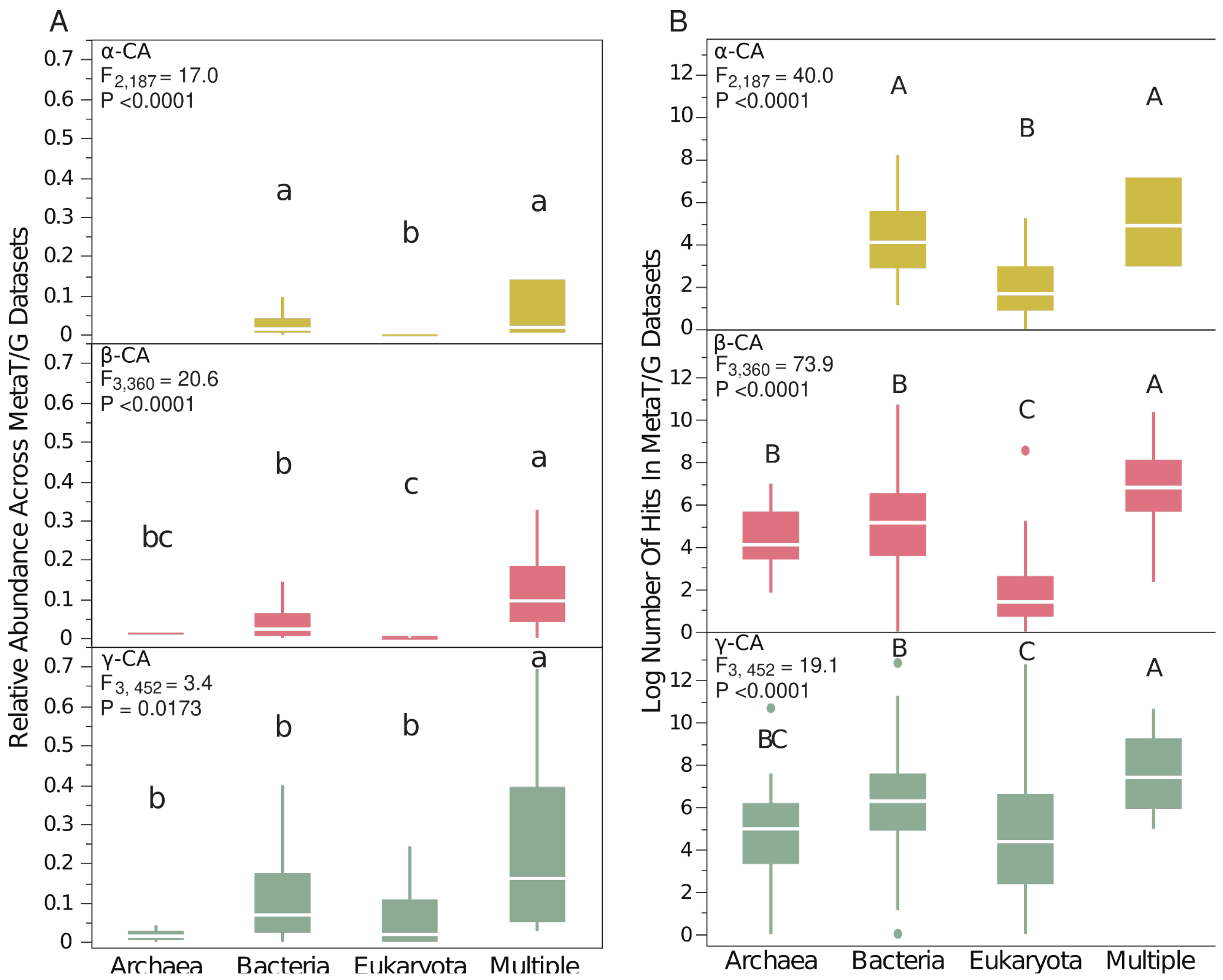

Figure S14. Median (**A**) relative abundance of α-, β-, and γ-CA clusters out of 3,672 metagenomic/metatranscriptomic datasets and (**B**) log number of total hits for each CA class as a function of the LCR taxonomic assignment of each CA. "Multiple" indicates CA clusters comprised of sequences from multiple Superkingdoms. Statistical results from one-way ANOVA for each CA class are shown in the upper left of each panel; letters correspond to differences after post-hoc Tukey's HSD ($P<0.05$). Quantile box plots show the minimum and maximum values (vertical lines), the 50th percentile (i.e., median; white line), and the 25th to 75th percentiles (area of the box).

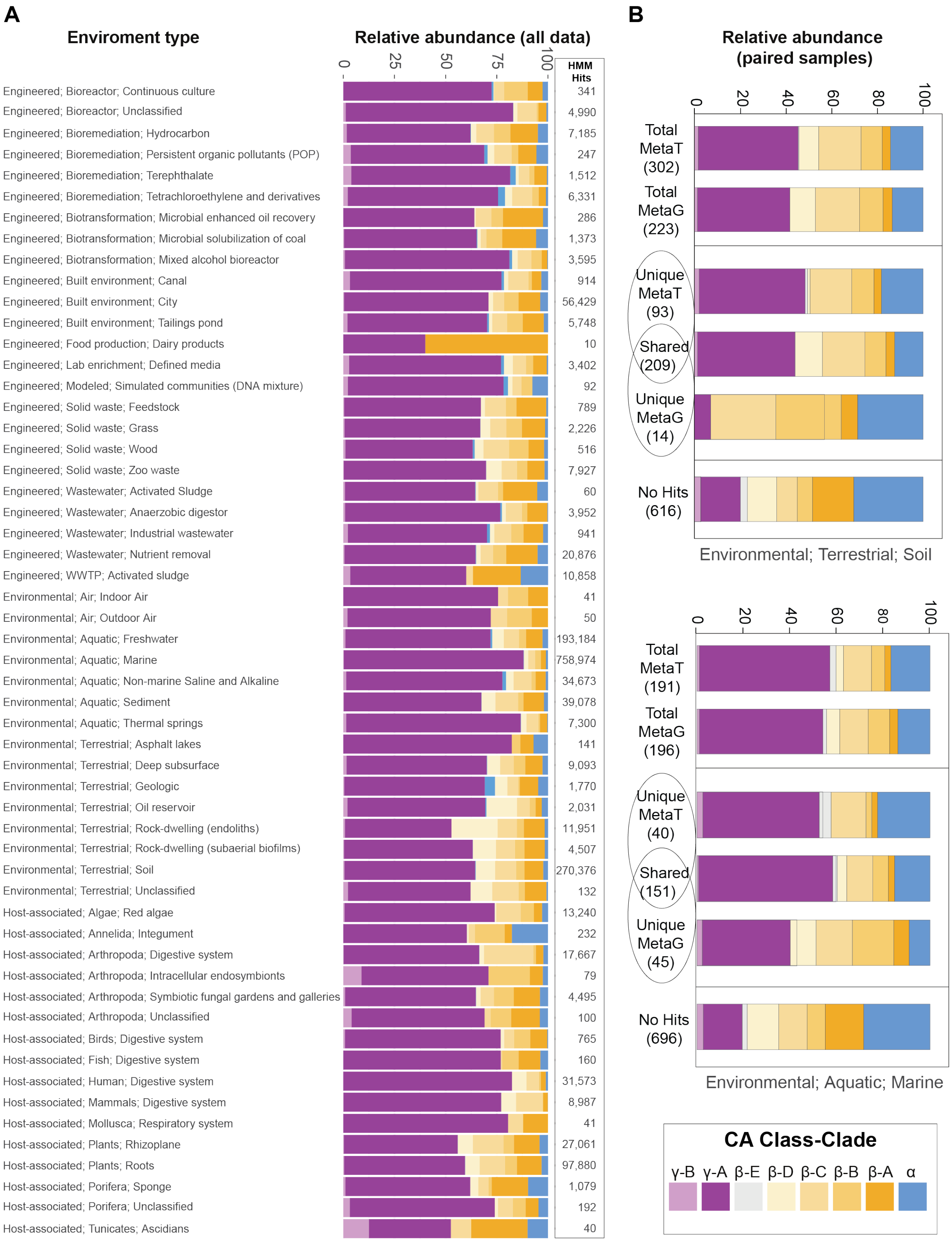
A
Enviroment type
Relative abundance (all data)
0
25
50
75
100
HMM Hits
Engineered; Bioreactor; Continuous culture 341
Engineered; Bioreactor; Unclassified 4,990
Engineered; Bioremediation; Hydrocarbon 7,185
Engineered; Bioremediation; Persistent organic pollutants (POP) 247
Engineered; Bioremediation; Terephthalate 1,512
Engineered; Bioremediation; Tetrachloroethylene and derivatives 6,331
Engineered; Biotransformation; Microbial enhanced oil recovery 286
Engineered; Biotransformation; Microbial solubilization of coal 1,373
Engineered; Biotransformation; Mixed alcohol bioreactor 3,595
Engineered; Built environment; Canal 914
Engineered; Built environment; City 56,429
Engineered; Built environment; Tailings pond 5,748
Engineered; Food production; Dairy products 10
Engineered; Lab enrichment; Defined media 3,402
Engineered; Modeled; Simulated communities (DNA mixture) 92
Engineered; Solid waste; Feedstock 789
Engineered; Solid waste; Grass 2,226
Engineered; Solid waste; Wood 516
Engineered; Solid waste; Zoo waste 7,927
Engineered; Wastewater; Activated Sludge 60
Engineered; Wastewater; Anaerzobic digestor 3,952
Engineered; Wastewater; Industrial wastewater 941
Engineered; Wastewater; Nutrient removal 20,876
Engineered; WWTP; Activated sludge 10,858
Environmental; Air; Indoor Air 41
Environmental; Air; Outdoor Air 50
Environmental; Aquatic; Freshwater 193,184
Environmental; Aquatic; Marine 758,974
Environmental; Aquatic; Non-marine Saline and Alkaline 34,673
Environmental; Aquatic; Sediment 39,078
Environmental; Aquatic; Thermal springs 7,300
Environmental; Terrestrial; Asphalt lakes 141
Environmental; Terrestrial; Deep subsurface 9,093
Environmental; Terrestrial; Geologic 1,770
Environmental; Terrestrial; Oil reservoir 2,031
Environmental; Terrestrial; Rock-dwelling (endoliths) 11,951
Environmental; Terrestrial; Rock-dwelling (subaerial biofilms) 4,507
Environmental; Terrestrial; Soil 270,376
Environmental; Terrestrial; Unclassified 132
Host-associated; Algae; Red algae 13,240
Host-associated; Annelida; Integument 232
Host-associated; Arthropoda; Digestive system 17,667
Host-associated; Arthropoda; Intracellular endosymbionts 79
Host-associated; Arthropoda; Symbiotic fungal gardens and galleries 4,495
Host-associated; Arthropoda; Unclassified 100
Host-associated; Birds; Digestive system 765
Host-associated; Fish; Digestive system 160
Host-associated; Human; Digestive system 31,573
Host-associated; Mammals; Digestive system 8,987
Host-associated; Mollusca; Respiratory system 41
Host-associated; Plants; Rhizoplane 27,061
Host-associated; Plants; Roots 97,880
Host-associated; Porifera; Sponge 1,079
Host-associated; Porifera; Unclassified 192
Host-associated; Tunicates; Ascidians 40
B
Relative abundance (paired samples)
0
20
40
60
80
100
Total MetaT (302)
Total MetaG (223)
Unique MetaT (93)
Shared (209)
Unique MetaG (14)
No Hits (616)
Environmental; Terrestrial; Soil
0
20
40
60
80
100
Total MetaT (191)
Total MetaG (196)
Unique MetaT (40)
Shared (151)
Unique MetaG (45)
No Hits (696)
Environmental; Aquatic; Marine
CA Class-Clade
γ-B
γ-A
β-E
β-D
β-C
β-B
β-A
α

Figure S15. Relative abundance of CA clades and classes for all environmental categories and for paired samples. Stacked bar chart showing the relative abundance of α-, β- and γ-CA HMM hits to (**A**) all metagenomic/metatranscriptomic database downloaded from IMG with environmental categories not collapsed, and (**B**) comparison of paired metagenomic and metatranscriptomic data for two environments. For panel A, numbers to the right of each bar represent the total number of CA HMM hits per environment for all CA classes. Although the greatest fraction of hits for each environment was to γ-CA, among the β-CAs, β-D CAs appear relatively abundant in terrestrial environments, particularly in rock-dwelling (endoliths) and soils. For panel B, numbers indicate the number of CA HMM clusters represented by all hits, and stacked bars indicate the relative abundance of all CA classes combined for each method (left side), CA that were either shared- or unique to a method (middle) or not observed in these samples (right side). CA clade information follows Fig. 4. Paired data are from IMG datasets: https://genome.jgi.doe.gov/portal/MetandMoHydrogen/MetandMoHydrogen.info.html and https://genome.jgi.doe.gov/portal/MicSysPacifOcean_2/MicSysPacifOcean_2.info.html

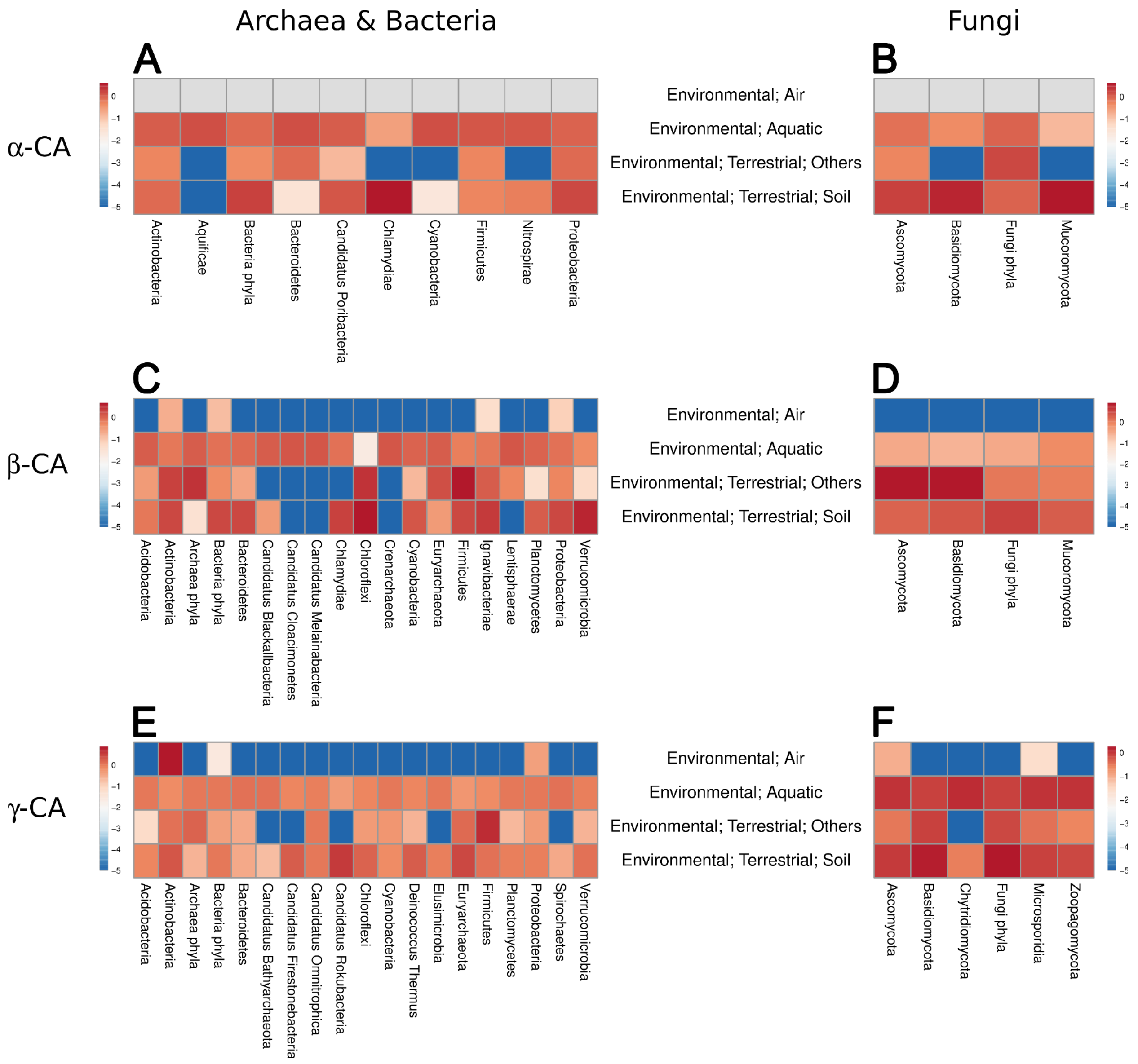

Figure S16. Heatmap showing the relative abundance of α-, β- and γ-CA HMM hits to the metaG/metaT database at IMG from different archaeal and bacteria or fungal phyla across the major environmental ecosystems for all HMMs. Archaeal and bacterial HMMs (a, c, e), and fungal HMMs (b, d, f) were subsampled and analyzed separately. The number of hits was scaled by the proportion of studies for the respective ecosystem and the abundance of each phylum and log transformed. Taxonomy was determined using LCR method. Grey color indicates no CA hits.

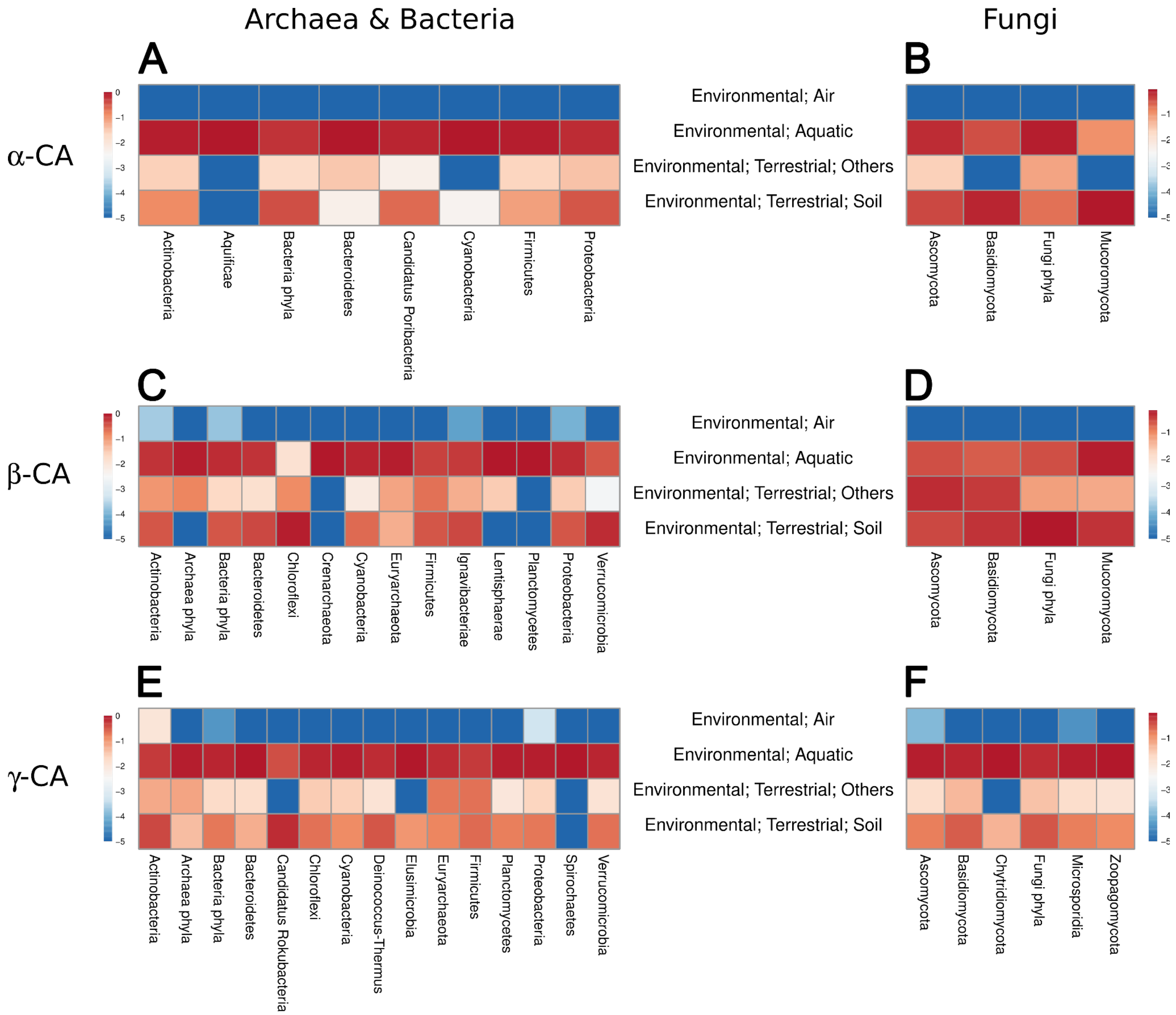

Figure S17. Heatmap showing the relative abundance of α-, β- and γ-CA HMM hits to the metaG/metaT database at IMG from different archaeal and bacteria or fungal phyla across the major environmental ecosystems for the ten most retrieved HMMs per ecosystem. Archaeal and bacterial HMMs (a, c, e), and fungal HMMs (b, d, f) were subsampled and analyzed separately. The number of hits was scaled by the abundance of each phylum, and log-transformed. Taxonomy was determined using LCR method. Grey color indicates no CA hits.

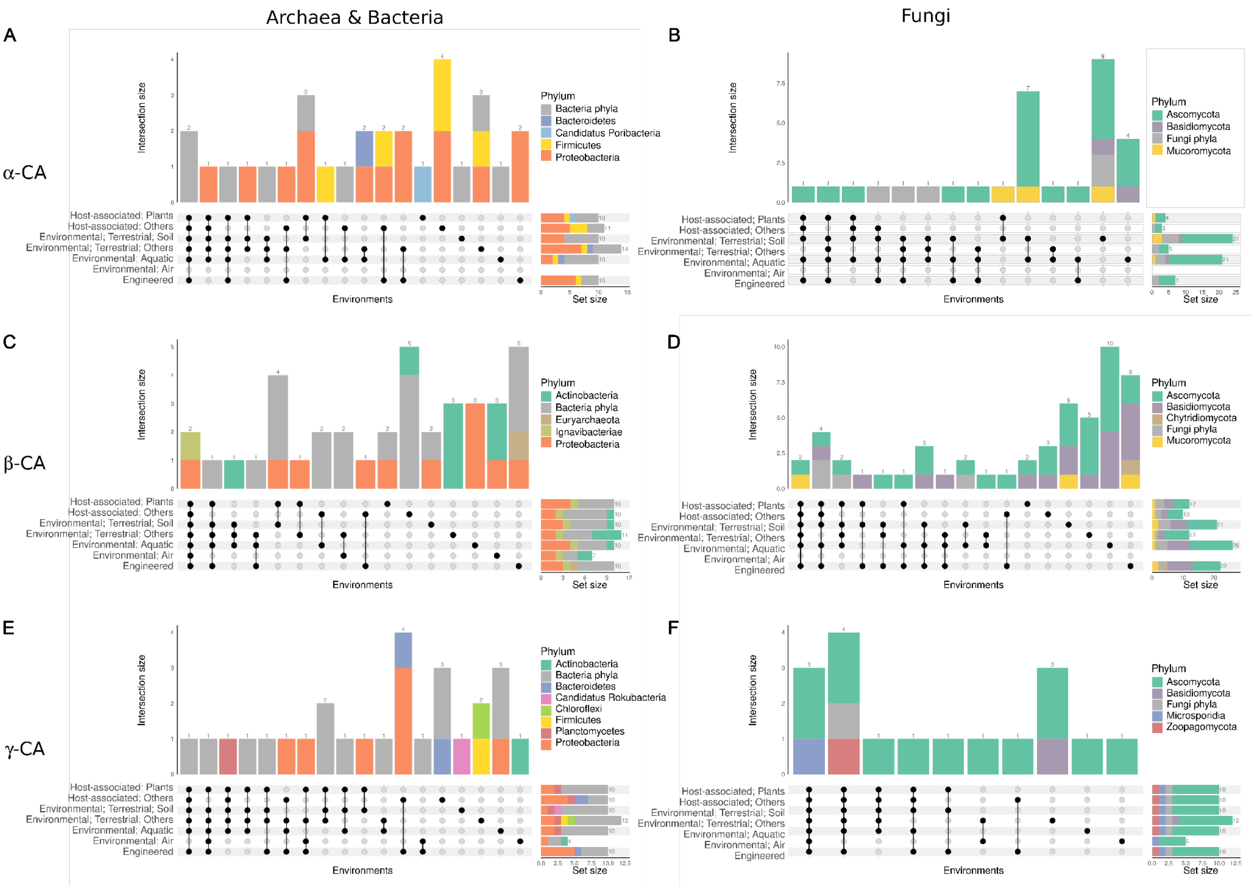

Figure S18. The intersection of different ecosystems for the 10 most retrieved HMMs per ecosystem. Archaeal and Bacterial HMMs (a, c, e), and Fungal HMMs (b, d, f) were subsampled and analyzed separately. Bars are colored by phyla, which was assigned by the LCR approach. See Fig. 6 for the contributions of all CA classes together. The most abundant HMMs are listed in Table S8.

ADDITIONAL FILES (available at Figshare: 10.6084/m9.figshare.28156742)

Additional file 1. Tab-separated file providing the identification, source, and taxonomy for the genes included in this study.

Additional file 2. Cluster FASTA-like format produced by MMseqs2 after clustering the sequences at 50% sequence similarity. Two successive identical names indicate a new cluster; the first sequence is the cluster representative sequence, which also gives the group its name.

Additional file 3. Tab-separated values (TSV) file showing the lowest taxonomic level shared by all the members in the cluster. The taxonomic ranks analyzed were superkingdom, kingdom, phylum, clade, class, order, family, genus, species, and strain.

Additional File 4. Edited multiple sequence alignments and corresponding phylogenetic trees for α-, β-, and γ-carbonic anhydrases (CAs).

Additional file 5. Count of CA HMM hits to the metaG/metaT database at IMG. GOLD Ecosystem classification is provided.

SUPPLEMENTAL REFERENCES

1. Steinegger M, Söding J. MMseqs2 enables sensitive protein sequence searching for the analysis of massive data sets. *Nat Biotechnol* 2017; **35**: 1026–1028.
2. Chamberlain SA, Szöcs E. taxize: taxonomic search and retrieval in R. *F1000Res* 2013; **2**: 191.
3. Winter D. rentrez: An R package for the NCBI eUtils API. *R J* 2017; **9**: 520.
4. Huson DH, Auch AF, Qi J, Schuster SC. MEGAN analysis of metagenomic data. *Genome Res* 2007; **17**: 377–386.
5. Lipscomb CE. Medical Subject Headings (MeSH). *Bull Med Libr Assoc* 2000; **88**: 265–266.
6. Liljas A, Laurberg M. A wheel invented three times. *EMBO Rep* 2000; **1**: 16–17.
7. Supuran CT. Structure and function of carbonic anhydrases. *Biochem J* 2016; **473**: 2023–2032.
8. Hewett-Emmett D, Tashian RE. Functional diversity, conservation, and convergence in the evolution of the alpha-, beta-, and gamma-carbonic anhydrase gene families. *Mol Phylogenet Evol* 1996; **5**: 50–77.
9. Smith KS, Jakubzick C, Whittam TS, Ferry JG. Carbonic anhydrase is an ancient enzyme widespread in prokaryotes. *Proc Natl Acad Sci U S A* 1999; **96**: 15184–15189.
10. Letunic I, Bork P. Interactive Tree Of Life (iTOL) v5: an online tool for phylogenetic tree

display and annotation. *Nucleic Acids Res* 2021; **49**: W293–W296.

11. Subramanian B, Gao S, Lercher MJ, Hu S, Chen W-H. Evolview v3: a webserver for visualization, annotation, and management of phylogenetic trees. *Nucleic Acids Res* 2019; **47**: W270–W275.
12. Carbone I, White JB, Miadlikowska J, Arnold AE, Miller MA, Magain N, et al. T-BAS version 2.1: Tree-Based Alignment Selector toolkit for evolutionary placement of DNA sequences and viewing alignments and specimen metadata on curated and custom trees. *Microbiol Resour Announc* 2019; **8**.
13. Meredith LK, Ogée J, Boye K, Singer E, Wingate L, von Sperber C, et al. Soil exchange rates of COS and CO18O differ with the diversity of microbial communities and their carbonic anhydrase enzymes. *ISME J* 2019; **13**: 290–300.
14. McLaren MR, Willis AD, Callahan BJ. Consistent and correctable bias in metagenomic sequencing experiments. *Elife* 2019; **8**.
